# Seasonal Variations in the Metabolome and Bioactivity Profile of *Fucus vesiculosus* Extracted by an Optimised, Pressurised Liquid Extraction Protocol

**DOI:** 10.3390/md16120503

**Published:** 2018-12-13

**Authors:** Edwin Heavisides, Caroline Rouger, Anna F. Reichel, Cornelia Ulrich, Arlette Wenzel-Storjohann, Susanne Sebens, Deniz Tasdemir

**Affiliations:** 1 GEOMAR Centre for Marine Biotechnology (GEOMAR-Biotech), Research Unit Marine Natural Products Chemistry, GEOMAR Helmholtz Centre for Ocean Research Kiel, Am Kiel-Kanal 44, 24106 Kiel, Germany; edheavisides@outlook.com (E.H.); caroline.rouger@u-bordeaux.fr (C.R.); reichel-anna@gmx.de (A.F.R.); cornelia.ulrich@mpimf-heidelberg.mpg.de (C.U.); awenzel-storjohann@geomar.de (A.W.-S.); 2 Institute for Experimental Cancer Research, Christian-Albrechts-University Kiel (CAU) and University Medical Center Schleswig-Holstein (UKSH) Campus Kiel, Arnold-Heller-Str. 3, Building 17, 24105 Kiel, Germany; susanne.sebens@email.uni-kiel.de; 3 Faculty of Mathematics and Natural Sciences, Christian-Albrechts-University Kiel (CAU), Christian-Albrechts-Platz 4, 24118 Kiel, Germany

**Keywords:** *Fucus vesiculosus*, bladder wrack, pressurised liquid extraction, definitive screening design, macroalga, untargeted metabolomics, seasonal variation, bioactivity

## Abstract

The metabolism of seaweeds depends on environmental parameters, the availability of nutrients, and biotic/abiotic stresses; therefore, their chemical composition fluctuates throughout the year. This study investigated seasonal variations in the metabolome of the Baltic Sea brown alga *Fucus vesiculosus* and its potential relation to the bioactivity profile. By using a definitive screening design (DSD) combined with pressurised liquid extraction (PLE), an optimised protocol was developed to extract algal biomass monthly for a full calendar year. An untargeted metabolomics approach using ultra-high performance liquid chromatography-tandem mass spectrometry (UPLC-MS^n^)-based molecular networking and manual dereplication was employed. The extracts were simultaneously screened for their in vitro antimicrobial, anticancer/apoptotic, and free radical scavenging activities. 44 compounds were putatively dereplicated in the metabolome. Many compounds were found to vary with the sampling month; phlorotannin total ion count (TIC) was highest in summer, whilst chlorophylls, lipids, and carotenoids peaked in winter and spring. The greatest radical scavenging and apoptotic activities against pancreas cancer cells observed in the summer months were attributed to high phlorotannin TIC. Methicillin-resistant *Staphylococcus aureus* (MRSA) inhibitory activity was produced year-round without a clear seasonal trend. This is the first study applying DSD-based optimised PLE extraction combined with a metabolome analysis of *F. vesiculosus* for the identification of seasonal variations in both metabolome and bioactivity.

## 1. Introduction

Seaweeds (marine macroalgae) are a diverse and ubiquitous group of eukaryotic, photosynthetic organisms that play essential roles in marine ecosystems. They are major primary producers that contribute to the marine carbon cycle and provide food and shelter to many aquatic animals [1]. The algal metabolism is dependent on environmental conditions, such as temperature, light, and nutrients that affect the production of metabolites as a response to such environmental conditions. Additional stresses, such as fouling, also show seasonal variations; hence, the chemical defense and overall metabolite profile of the seaweeds are subject to fluctuations during the year [2]. Brown algae (Phaeophytes) have been the focus of many studies because they are easy to access and are rich in various classes of natural products. These compounds, which are produced as an evolutionary response to environmental factors and pressures, also have pharmaceutical relevance to the treatment of human diseases [3]. Brown algae have indeed long been used in traditional medicine due to their wide-ranging biological activities and in the discovery of new drug leads [4]. However, the potential impact of seasonal variations in their metabolite and bioactivity profile is often neglected in analytical and drug discovery studies.

In recent years, the use of design of experiments (DoE) has become more common in the optimisation of the extraction of natural products. DoE involves the statistical prediction of the effect of changing independent input variables on dependent output variables. By identifying multiple input variables that are most important in the yield and quality of natural extracts (e.g., temperature, time, solvent volume), it is possible to create optimal extraction processes whereby the most important extraction variables are prioritised and wastage is greatly reduced. Various types of DoE use different statistical models and approaches and definitive screening designs (DSD) represent one of the latest generations of DoE. Using only three factor levels (low, medium, and high) for each input variable (*n*), a DSD is able to assess the individual effect of the input variables on the output variables in only 2*n*+1 experimental runs. Traditional ‘one-variable-at-a-time’ optimisation strategies are often inefficient in time, solvents, and biomass. Hence, the reduction of the necessary optimisation runs through sophisticated screening designs can greatly speed up the extraction process and preserve biological material for further studies [5]. Although not frequently employed in the optimisation of natural product extraction, DSDs have been successfully employed in biological systems to optimise mass spectrometry parameters [6], therapeutic protein treatment [7], and bioreactor fermentation [8].

Traditional extraction techniques for brown algae generally consist of maceration [9]; i.e., agitation of the algal material in solvents for a large number of hours at temperatures ranging from ambient to 50–60 °C [10,11]. Commonly used solvents include methanol [12], ethyl acetate (EtOAc) [13], dichloromethane [14], and ethanol [15] or their mixtures. As previous studies have mostly focused on specific brown algal metabolites rather than analysing the whole metabolome, the solvent of choice, extraction temperatures, and extraction time vary in the published literature. However, these non-automated techniques are time-consuming and suffer from low reproducibility; hence, they are unsuitable for metabolomics studies. Pressurised liquid extraction (PLE) is recognised as an eco-friendly technique, ensuring efficient yield recoveries with a short extraction time and reduced solvent consumption. It uses elevated temperatures and pressures to maximise the solubility of natural compounds, and has the advantage of being fully automated, increasing reproducibility. The possibility for successive extractions using a variety of solvents enables the recovery of a wide range of metabolites, whose rate can be optimised by adjusting the PLE parameters [16]. The optimisation of the extraction of specific chemical classes using PLE has successfully been implemented in the extraction of fucoxanthin from the brown alga *Eisenia bicyclis* [17] and fatty acids from a range of brown and green algae, including *F. vesiculosus* [18]. However, the use of statistical DoE to optimise a general PLE procedure for studying the brown algal metabolome has not been reported so far.

*Fucus vesiculosus*, also known as bladder wrack, is a foundation species in the littoral zone of rocky shore ecosystems around Northern Europe and the Atlantic coast of North America. Bladder wrack is used extensively in the pharmaceutical, nutraceutical, and cosmetics industries due to its high fiber, iodine, and bioactive natural product contents [19]. The best-known natural product classes from *F. vesiculosus* include phlorotannins, fucoxanthin, and fucoidans. Phlorotannins, the polymers of phloroglucinol, are structural components of brown algal cell walls [20]. These polyphenols protect the alga against harmful ultraviolet (UV) radiation, and deter predation through their bitter taste [21]. Phlorotannins also show relevant biological activities, including antioxidant, anticancer, and antimicrobial activities [22,23]. The distinctive brown colouration of *Fucus* spp. is due to the high abundance of phlorotannins and also the high concentration of the carotenoid fucoxanthin [24]. The photosynthetic pigment fucoxanthin is a strong free radical scavenger with antioxidant as well as anticancer, antidiabetic, antimalarial, and antiobesity activities [25,26,27]. Fucoidan and other sulphated polysaccharide polymers present in *F. vesiculosus* have been the subject of many studies [28,29,30]. Despite the limited data available on the environmentally controlled regulation of the production of fucoidan by *F. vesiculosus* [31], variation in seasonal fucoidan levels has been observed in multiple brown algal species [32], indicating a response to environmental changes. A previous gas chromatography coupled to mass spectrometry (GC-MS)-based study investigated the seasonal composition of the surface-associated metabolites in *F. vesiculosus* [33]; however, seasonal variations in the whole *F.vesiculosus* metabolome have never been studied.

This study aimed to identify seasonal changes in the metabolite and bioactivity profiles of Baltic Sea *F. vesiculosus* sampled monthly for a full calendar year and seek possible correlations between bioactivity and variations in metabolite composition. A DSD-based approach was used to develop an optimised PLE protocol based on six water-extraction-independent variables and five organic solvent extraction-independent variables using the Accelerated Solvent Extraction (ASE) system for extraction of the algal biomass. Previous studies have reported antibacterial and antifungal activities by *F. vesiculosus* extracts [3]. On this basis, bacterial and fungal pathogen strains relevant to pharmaceutical research were selected for bioassay screening, including the ESKAPE panel of human bacterial pathogens (*Enterococcus faecium*, methicillin-resistant *Staphylococcus aureus* (MRSA), *Klebsiella pneumoniae*, *Acinetobacter baumannii*, *Pseudomonas aeruginosa*, and *Escherichia coli*), various environmental pathogens (*Vibrio anguillarum*, *Pseudoalteromonas bacteriolytica*, and *Pseudoalteromonas elyakovii),* and two human fungal pathogens (*Candida albicans* and *Cryptococcus neoformans*). *F. vesiculosus* extracts have also been shown to effectively scavenge free radicals [34]; this was tested in the present study using a rapid 2-diphenyl-1-picrylhydrazyl (DPPH) free radical assay. Finally, the potential anticancer activity of the *F. vesiculosus* extracts was tested against A-549 lung carcinoma cells, MB-231 breast carcinoma cells, and Panc1 human pancreatic ductal adenocarcinoma cells. The latter was previously shown to be inhibited by *F. vesiculosus* crude extracts and fractions [35,36]. Using the combination of a multi-variables DSD with PLE, monthly *F. vesiculosus* samples from a whole calendar year were subjected to untargeted metabolomics and bioactivity screenings to assess the potential effect of season on the presence and bioactivity of pharmacologically relevant molecules.

## 2. Results

### 2.1. Optimisation of the PLE Conditions

Extraction of the freeze-dried and ground *F. vesiculosus* samples consisted of two consecutive steps: a preliminary water extraction step (to basically remove the sea salts that may interfere with the subsequent sensitive ultra-high performance liquid chromatography-mass spectrometry (UPLC-MS) analyses), followed by subsequent extractions with 100% methanol (MeOH) and 100% dichloromethane (DCM).

#### 2.1.1. The Water Extraction Step

The success of the water extraction step was measured using yield as a response variable; higher yield values indicated the removal of greater amounts of undesired salts from the *Fucus* samples. For the DSD, six experimental variables (temperature, number of static cycles, static time, rinse volume, sample weight, and ratio of sand:*Fucus vesiculosus* material, denoted *Fucus* from here onwards) were selected to test for significant effects on yield. Each variable was also assigned three factor levels (low, medium, and high). A DSD matrix produced a sequence of 13 different combinations of the factor levels for each of the six experimental variables. The combination of low, medium, or high factor levels was calculated to provide a comprehensive analysis of the significance of each experimental variable’s significance. Four of the experimental variables, namely temperature (20, 40, 60 °C), number of static cycles (1, 2, 3), static time (5, 8, 11 min), and rinse volume (30, 75, 120% of cell) are the main programmable variables with the ASE 350™ system. The other two experimental variables related to the set up of the extraction cells were sample weight (0.5, 1.0, 1.5 g) and ratio of sand:*Fucus* (10:1, 15:1, 20:1). The three levels for each variable were chosen to preserve the chemical composition, to keep the extraction time within reasonable limits, or to minimize the necessary solvent and biomass usage.

**Figure 1:**
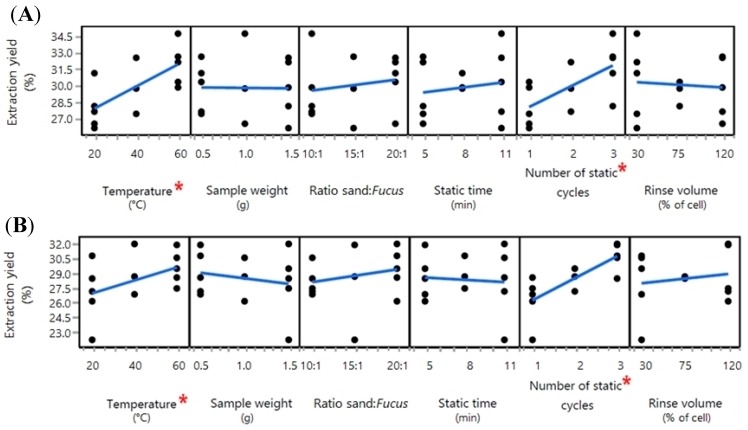
Statistical regression analyses of the relationship between extraction yield of *Fucus vesiculosus* and the six extraction variables (temperature, sample weight, ratio of sand:*Fucus* algal material, static time, number of static cycles, and rinse volume) of the water extraction step’s first (**A**) and second (**B**) repeat. Significant regressions are denoted by *.

The 13-run DSD sequence was repeated twice to compare the reproducibility of the results. Of the six extraction variables tested in the first repeat of the water extraction DSD sequence, temperature and number of static cycles were calculated to be the main effect variables based on a root-mean-square analysis of linear regression models (Figure 1A). In both cases, a positive relationship was found between the extraction yield and the extraction variable within the tested experimental ranges. The second repeat of the water extraction DSD sequence produced comparable results to the first repeat (Figure 1B). Each repeat indicated that the temperature and the number of static cycles had the most significant positive effect on extraction yield. 

The results of the two concurrent repeats of the water extraction DSD matrices were integrated into an optimised water extraction step to be run before each subsequent organic solvent extraction. As the aim of the extraction process was to study the largest possible part of the metabolome, the temperature was limited to 40 °C to avoid the potential thermal degradation of molecules. Although PLE is capable of effectively extracting compounds at high temperatures, this is only possible with shorter extraction times. However, in the present DSD design, total extraction times, including for the water, MeOH, and DCM stages ranged from 15 to 99 min depending on the DSD run. Previous studies have found that lower temperatures (25–55 °C) over longer extraction times (2–24 h) for both PLE and other extraction techniques can maximise phlorotannin [9], carotenoid [11], and terpene yield [16], in addition to antioxidant activity [10] in extracts from various brown macroalgae. The number of static cycles was set to 3. Lower ratios of sand:*Fucus* material were observed to have a high rate of cell blockage errors during the cell rinse phase. Therefore, the ratio sand:*Fucus* material was set to 20:1. The cell rinse volume was also limited to 30% to minimise cell blockage. Within the tested experimental range, these three variables were not found to have a significant effect on yield, and could, therefore, be set to the above levels. Similarly, static time was set to the high experimental factor level of 10 min, despite not being a statistically significant main effect variable. Although this increased the overall extraction time, it was taken as a precaution as water extraction steps with shorter static cycle times produced viscous final organic solvent extracts with high amounts of cell wall polysaccharides.

#### 2.1.2. The Organic Solvent Extraction Step

MeOH and DCM were selected due to their broad polarity coverages and their frequent use in exhaustive extraction of algal metabolites [37,38]. For optimisation of the extraction step using MeOH followed by DCM, five input variables with three factor levels were selected. The five extraction steps were temperature (20, 40, 60 °C), number of MeOH and DCM static cycles (both 1, 2, 3), and time of the MeOH and DCM static cycles (both 5, 8, 11 min). Rinse volume was not changed for this step, as high rinse volumes were observed to increase cell blockage rates. The set up of the extraction cell preparation was established during the previous water extraction step and could, therefore, not be used as variable in the organic solvent step.

For further analyses, the MeOH and DCM extracts were combined to reduce the number of experimental and analytical steps whilst still investigating the full metabolome. To evaluate the chemical diversity of the combined MeOH:DCM extracts, the number and area of peaks in high-performance liquid chromatography (HPLC)-UV (280 and 405 nm detections) and HPLC- evaporative light scattering detector (ELSD) chromatograms, as well as the number of nodes and clusters in molecular networks generated from HPLC-MS^n^ data with the Global Natural Products Social Molecular Networking (GNPS) online platform [39], were used as output variables. These molecular networks are represented by clusters of compounds (nodes) connected together based upon similarities of mass fragmentation (MS/MS) patterns. The variation between the results of the two organic solvent extraction DSD matrix repeats was much higher than those of the water extraction optimisation stage. A DSD analysis of the matrix repeats found no concurrent effect of any of the input variables on any of the recorded measures of chemical diversity. Linear regression plots indicating the significant main effect variables on any of the chemical diversity indices in only one of the two repeats are shown in the supplementary data (Figure S1).

**Figure 2:**
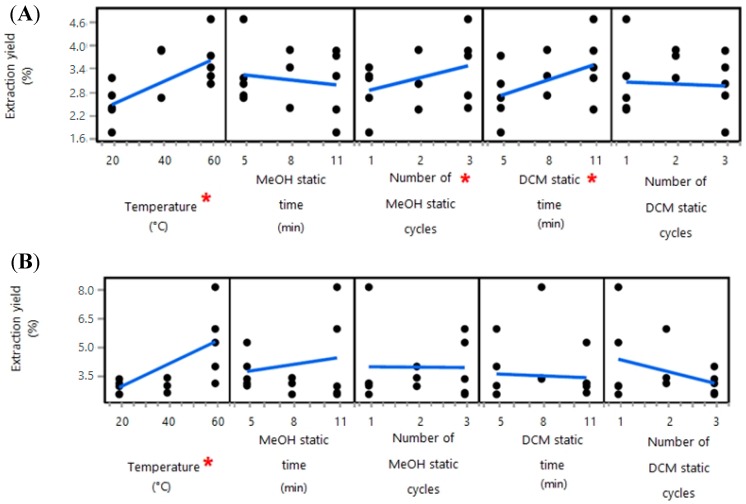
Linear regressions of the relationship between the extraction yield and the five extraction variables (temperature, MeOH static time, number of MeOH static cycles, DCM static time, and number of DCM static cycles) of the organic solvent extraction step’s first (**A**) and second (**B**) repeat. Significant regressions are denoted by *.

The only significant main effect variable that was shared between the repeat analyses was the temperature, which in both cases was found to have a significant positive relationship with extraction yield (Figure 2). For the subsequent extractions of the *F. vesiculosus* sample collected monthly, the temperature was limited to 40 °C to avoid potential thermal degradation of compounds and to remain consistent with the water extraction step. All other variables tested during the organic solvent extraction optimisation stage were set to the most time- and resource-efficient levels within the experimental range, namely 5 min MeOH static time, one MeOH static cycle, 5 min DCM static time, and one DCM static cycle.

### 2.2. Analysis of the Monthly Extracts

The yields of the water and organic solvent extracts were recorded to compare the amount of specific solvent-soluble compounds from the samples of *F. vesiculosus* collected each month. The organic solvent extracts were further analysed using UPLC-quadrupole time-of-flight (QTOF)-MS/MS to dereplicate specific compounds and identify chemical fluctuations between the months and seasons. The same extracts were also tested against a panel of pathogenic microorganisms and cancer cell lines to assess any variations in their bioactivity profiles. An LC-MS analysis was not performed on the *F. vesiculosus* water extracts due to the presence of salts, which may have interfered with the highly sensitive UPLC-MS instrument. Similarly, the water extracts were not tested for bioactivity due to the high concentrations of salts and primary metabolites, which may have affected culture growth rates.

#### 2.2.1. The Yield of Water and Organic Solvent Extracts

**Figure 3:**
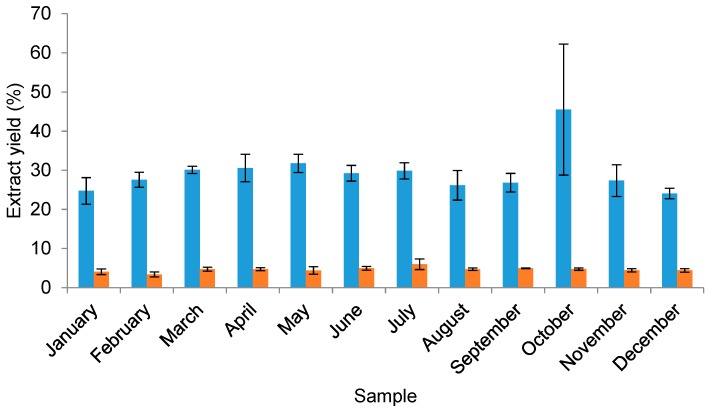
The yields (%) obtained with water (blue) and organic solvent (orange) PLE extracts of monthly *F. vesiculosus* samples from January 2017 to December 2017. Error bars indicate the standard deviation.

All dried water and organic solvent extracts were weighed to determine monthly yields (Figure 3). Yields obtained from water extractions accounted for a large percentage of the dried *F. vesiculosus* samples. The lowest mean yield obtained was 24.05 ± 1.34% from the December samples, compared to the highest yield of 45.50 ± 16.75% from the October samples. Despite the range in yield values, an analysis of the data using a Kruskal–Wallis test did not indicate a significant effect of collection month on mean water extract yield (H = 18.18, *p* = 0.077). The mean organic solvent extract yield ranged from 3.38 ± 0.65% (February samples) to 5.97 ± 1.35% (July samples). One-way ANOVA testing indicated a significant difference among the yields from the different collection months (*F*(11, 24) = 2.705, *p* = 0.02). A further analysis with Tukey’s pairwise tests indicated a significant difference in obtained organic solvent extract yields between July (mean (M) = 59.7, standard deviation (SD) = 13.5) and both January (M = 40.7, SD = 7.3) and February (M = 33.8, SD = 6.5). These significant differences suggest that the concentration of organic solvent-soluble compounds is higher in the middle of summer compared to winter.

#### 2.2.2. Metabolome Profiling of the *F. vesiculosus* Organic Solvent Extracts

**Figure 4:**
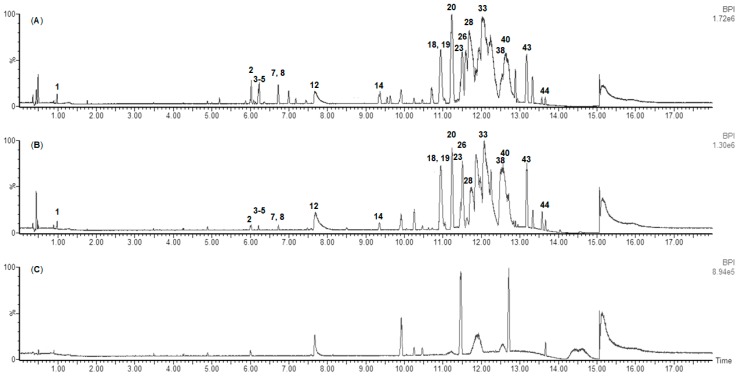
Positive ion mode UPLC-MS base peak ion (BPI) chromatograms with numbers highlighting the major recurring compounds present in the crude *F. vesiculosus* extracts obtained from (**A**) February 2017 and (**B**) August 2017 samples; (**C**) is an extraction blank for reference.

**Figure 5:**
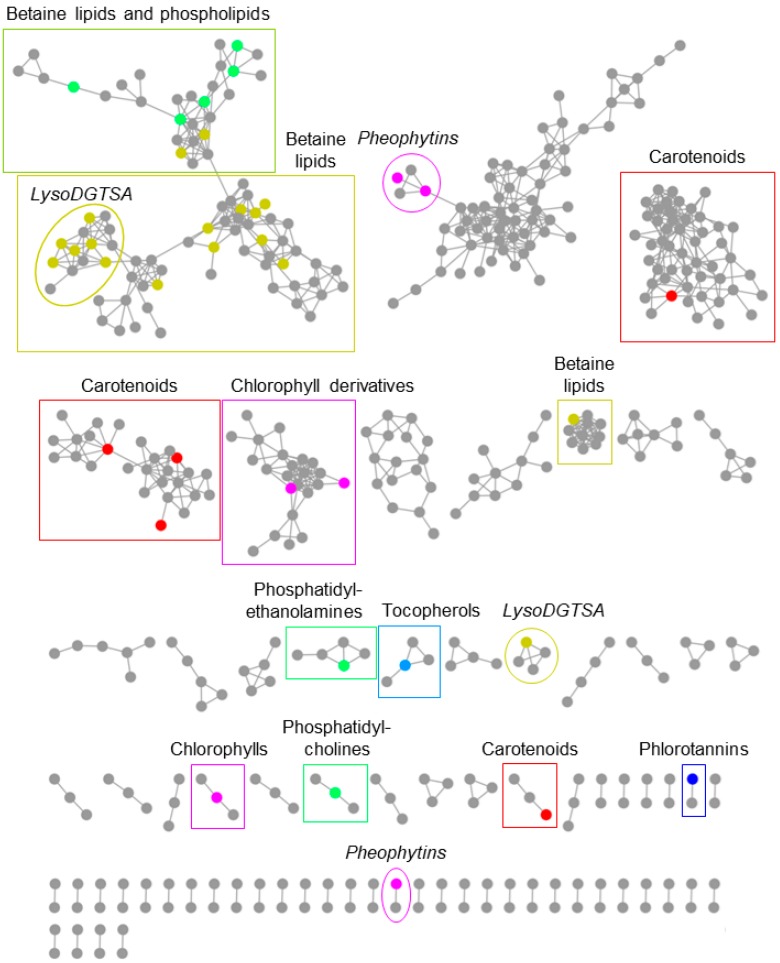
A UPLC-MS^2^-based molecular network showing the compounds present in the January–December organic algal extracts. Putatively dereplicated compounds are indicated by the following colour-coding: yellow = betaine lipids; red = carotenoids; pink = chlorophylls; green = phospholipids; light blue = tocopherols; dark blue = phlorotannins. Metabolites subclasses are indicated in italics. Clusters are putatively identified as these chemical (sub)classes based on the constituent dereplicated compounds. Chemical classes may be represented by multiple clusters due to limitations in the GNPS parameters and the presence of common fragmentation patterns.

UPLC-MS/MS data were obtained for each organic extract replicate for the monthly samples from January 2017 to December 2017. Chromatograms were manually analysed, and 20 major recurring peaks were identified in extracts from all collection months. Figure 4 shows that these peaks are present in two *F. vesiculosus* extracts sampled six months apart: February 2017 and August 2017. Between 11 and 14 min, clear peak separation was not achievable; therefore, peak density and overlap in this area of the chromatograms are high, and only the most intense peaks were annotated. The highlighted compounds were similarly detected in all other monthly extracts, which indicates their ubiquity. The analysis of the UPLC-photodiode array (PDA)-MS^2^ data enabled the putative dereplication of 44 compounds present in *F. vesiculosus* extracts. As mentioned above, 20 compounds were the major recurring compounds identified in every chromatogram of the algal samples collected monthly, whilst the other 24 were minor and not always present (Table S1). In addition to manual dereplication, UPLC-MS^2^ data were uploaded to the GNPS online platform [39] to generate a molecular network of the compounds present in the monthly extracts (Figure 5). Nodes representing individual compounds are clustered together based on the similarity of fragmentations observed in the MS/MS spectra. This allowed for putative identification of chemical classes amongst the node clusters, aiding dereplication of the recurring peaks.

**Figure 6:**
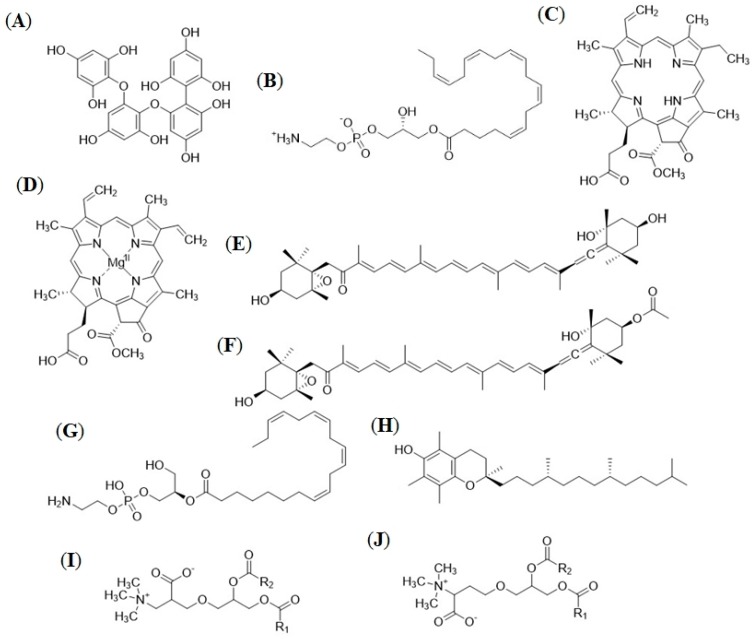
The chemical structures of: (**A**) Fucodiphlorethol phlorotannin; (**B**) LysoPC 20:5; (**C**) Pheophorbide *a*; (**D**) Chlorophyll *c2*; (**E**) Fucoxanthinol; (**F**) Fucoxanthin; (**G**) LysoPE 20:4; (**H**) α-Tocopherol; (**I**) DGTA’s general structure; (**J**) DGTS’s general structure.

The 20 major compounds identified could be classified into five chemical classes, namely phlorotannins (tetramer; compound **1**; Figure 6A), phosphatidylcholine (lysophosphatidylcholine 20:5; compound **2**; Figure 6B), betaine lipids and their lyso derivatives (compounds **3**–**5**, **7**, **8**, **25**, **28**, **33**, and **38**), chlorophylls (compounds **14**, **20**, **40**, and **43**; Figure 6C,D), and carotenoids (compounds **12**, **18**, **19**, and **44**; Figure 6E,F). Compounds putatively belonging to the classes of phosphoethanolamines (Figure 6G) and tocopherols (Figure 6H) were further dereplicated amongst the minor metabolites. Lipids were the most abundant of the identified compound classes, accounting for over half (29 out of 44) of the total dereplicated compounds, with 19 of these being identified as betaine or lyso-betaine lipids. Differentiation between 1,2-diacylglyceryl-*O*-2′-(hydroxymethyl)-(*N*,*N*,*N*-trimethyl)-β-alanine (DGTA; Figure 6I) and 1,2-diacylglyceryl-*O*-2′-(hydroxymethyl)-(*N*,*N*,*N*-trimethyl)homoserine (DGTS; Figure 6J) structural isomers could not be accomplished with the analytical techniques used in this study; therefore, all putative betaine lipids are referred to as DGTSA or lyso-DGTSA. The other 10 lipids identified all belonged to either phosphatidylethanolamine or phosphatidylcholine phospholipid subclasses. Chlorophyll and carotenoid pigments and their derivatives were also key classes with seven and six compounds identified, respectively. This included fucoxanthin, which was detected in both its intact (compound **19**) and dehydrated (compound **12**) forms. Table S1 shows the detailed dereplication of the compounds.

All five chemical classes were clearly identified in the molecular network (Figure 5). Phospholipids and betaine lipids accounted for the major clustering classes present, supporting the dominance of lipids in the dereplicated major and minor compounds. Chlorophyll compounds were the next most abundant in the molecular network in terms of the number of clusters (four); however, the three carotenoid clusters accounted for a higher total of nodes. Phlorotannins and tocopherols were present in the molecular network only as very minor compounds; each chemical class was only represented by one cluster, with four nodes comprising the tocopherol cluster and two for the phlorotannin cluster. Chemical classes may be represented by multiple clusters due to limitations in the GNPS parameters and the presence of common fragmentation patterns. All clusters were present in all monthly samples, and there were no chemical classes or fragmentation groups specific to a particular month or season.

#### 2.2.3. Seasonally Varying Compounds in the *F. vesiculosus* Extracts

A statistical analysis of the UPLC-MS data identified numerous major compounds for which the sampling month had a statistically significant effect on the mean peak intensity. Total ion count (TIC) data were filtered for all the compounds detected in the monthly samples by the XCMS Online platform [40] to include only compounds that exhibited significant TIC (≥100,000) in at least one of the monthly chromatogram replicates. A total of 54 compounds (TIC ≥ 100,000) were found by one-way ANOVA testing to show a significant effect of sampling month on TIC. The 54 compounds were separate from those identified as part of the dereplication study; however, 31 of the 54 compounds were already putatively identified as part of the overall metabolome (see Section 2.2.2.). The other 23 compounds could not be identified using the public and commercial libraries used in this study, suggesting that they may potentially be new compounds. These 54 compounds showing significant variation in TIC with sampling month were classified into the following chemical groups: one phlorotannin (tetramer), four carotenoids, five chlorophylls, 14 betaine lipids, and seven phospholipids. The retention time and *m*/*z* data for the remaining 23 compounds can be found in the supplementary data (Table S2).

The TIC profiles of the 31 significantly variable compounds from the samples collected monthly consisted of five general repeating ‘seasonality patterns’. These five patterns approximately corresponded to the chemical groups of phlorotannin, chlorophyll, carotenoid, and lipid (two contrasting patterns: one winter peaking and one summer peaking). The only phlorotannin tetramer putatively identified within the extracts (**1**) exhibited a seasonality pattern unique to itself. The TIC for this metabolite was lowest in the winter months with a noticeable late spring peak in May. Chlorophylls and their derivatives, including pheophorbides, generally exhibited peak abundance during the winter months with an annual low for the duration of the summer months, June, July, and August. The autumnal rate of recovery was much more pronounced for pheophorbide *a* (**20**) and pheophytin *c2* (**23**), whereas it was relatively lower for methylpheophorbide *a* (**40**). The general pattern displayed by the chlorophyll derivatives, however, was a winter peak followed by a summer trough. Similar to the chlorophyll pigments, carotenoids were most abundant during the winter months. The time period for which the TIC of the chlorophyll and carotenoid pigments is at its lowest is the key difference between the two seasonality patterns. Whilst chlorophylls were at their lowest for most of the summer, carotenoids presented a very typical May trough followed by recovery in the summer.

In the lipid class, there were two contrasting seasonality patterns. The dominant pattern was similar to that of the chlorophylls; the TIC was highest during the winter and spring months and fell to its lowest point during the summer. However, the non- or mono-unsaturated betaine lipids DGTSA 28:0 (14:0/14:0; **31**), DGTSA 30:0 (16:0/14:0 or 14:0/16:0; **38**), and DGTSA 32:1 (18:1/14:0 or 14:0/18:1; **39**) presented the opposite pattern; i.e., in these cases, the TIC was highest in late summer and autumn, while it was lowest in the winter and spring months. The differences in seasonal abundance could not be attributed to differences in lipid subclass. All three compounds exhibiting the contrary seasonal pattern were putatively identified as betaine lipids; however, most other betaine lipids exhibited the predominant winter/spring peak pattern. The degree of unsaturation did not appear to affect the observed seasonality pattern. All lyso-betaine lipids and other phospholipids also had peak TIC levels in winter and spring.

The remaining unidentified 23 compounds exhibited generally similar seasonal profiles. Peak levels were generally similar during the winter months, with the lowest levels in late spring and summer, similar to the chlorophyll, carotenoid, and one of the lipid patterns. Monthly TIC box plots for all identified and unidentified significantly variable compounds can be found in the supplementary data (Figures S2–S8). Thirteen of the 23 compounds exhibited a maximum TIC of over 1,000,000; this indicates that they may be the major components of the *F. vesiculosus* metabolome.

### 2.3. Bioactivity Screening

#### 2.3.1. Effect of Organic Extracts on Pathogenic Microorganisms

Each *F. vesiculosus* organic extract was tested against an array of pathogenic microbes of interest to drug discovery. Human pathogens tested consisted of the pathogenic yeast strains *Candida albicans* and *Cryptococcus neoformans*, and the ESKAPE panel of nosocomial bacteria, i.e., *Enterococcus faecium*, MRSA, *Klebsiella pneumoniae*, *Acinetobacter baumannii*, *Pseudomonas aeruginosa*, and *Escherichia coli*. Also tested were the three environmental pathogens *Pseudoalteromonas bacteriolytica*, *Pseudoalteromonas elyakovii*, and *Vibrio anguillarum*, known to affect seaweeds and fish species.

**Figure 7:**
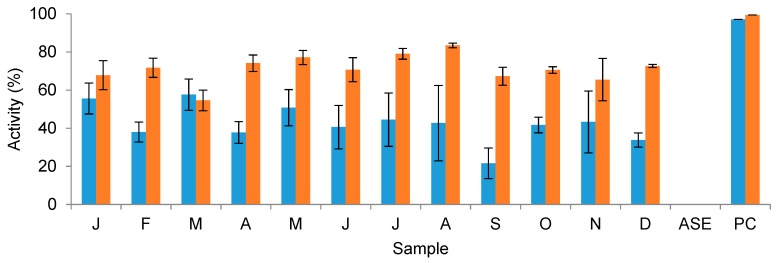
Anti-MRSA (blue) and DPPH free radical scavenging (orange) activities (%) of the monthly *F. vesiculosus* organic solvent extracts at a test concentration of 100 µg/mL. Also shown are activities of the ASE extraction blanks and the positive controls (PC) chloramphenicol (MRSA) and ascorbic acid (DPPH). Error bars indicate the standard deviation.

By applying a 20% threshold to indicate significant activity, only MRSA displayed a mean monthly inhibition by the crude *F. vesiculosus* extracts at a concentration of 100 μg/mL (Figure 7) compared to the DMSO negative control. Mean inhibition for all other strains fell below the 20% inhibition threshold. Mean inhibition of MRSA for all collection months was above the 20% threshold; the lowest mean inhibition was that of September (21.6%), whereas the highest mean inhibition was observed in the March extracts (57.7%). Mean anti-MRSA activity, including standard deviations, can be found in Figure 7. A one-way ANOVA test showed that there was a significant effect of sampling month on MRSA inhibition (*F*(11, 24) = 2.577, *p* = 0.025). Tukey’s pairwise post-hoc testing indicated that the level of inhibition from extracts obtained from September samples (21.6%) was significantly lower than those of March (57.6%).

#### 2.3.2. DPPH Free Radical Scavenging Activity

An assessment of the antioxidant activity of the crude *F. vesiculosus* organic extracts using the DPPH assay indicated the high DPPH free radical scavenging activity of all monthly samples at a concentration of 100 μg/mL. Furthermore, a one-way ANOVA test showed that there was a significant effect of sampling month on antioxidant activity (*F*(11, 24) = 5.818, *p* = < 0.001). Figure 7 shows the mean inhibition, including standard deviation, of the DPPH free radicals by each collection month. Tukey’s pairwise post-hoc testing indicated a significant annual peak in August (83.4%), compared to the lowest levels of inhibition exhibited by the March extracts (54.6%). Generally, DPPH free radical scavenging activity levels were at their lowest in early spring, before increasing rapidly towards their peak in late summer, followed by a steady decline in the winter months.

#### 2.3.3. Growth Inhibitory Activity on Cancer Cell Lines

**Figure 8:**
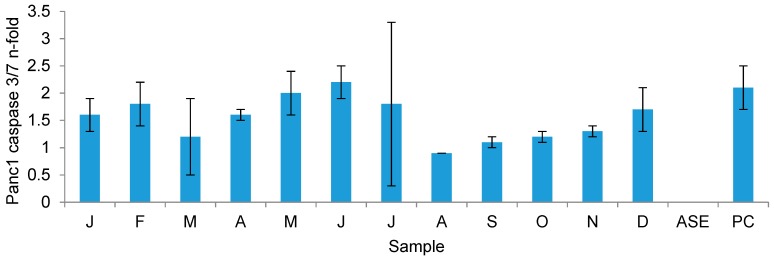
Panc1 caspase 3/7 *n*-fold activities of the monthly *F. vesiculosus* organic solvent extracts at a test concentration of 100 µg/mL. Also shown are the activities of the ASE extraction blanks and the positive control gemcitabine. Error bars indicate the standard deviation.

The monthly algal organic extracts were tested for growth inhibitory activity against A-549 lung carcinoma and MB-231 breast carcinoma cells, as well as for general toxicity against human keratinocyte (HaCaT) cells. The human pancreatic ductal adenocarcinoma cell line Panc1, which has already been used in former studies with *F. vesiculosus* extracts [35,36], was also included to test the cytotoxic activity of monthly extracts. To analyse whether monthly algal extracts exert pro-apoptotic effects, Panc1 cells were exposed to crude extracts at a concentration of 100 μg/mL. After 24 h, activity of the effector caspases-3/-7 was determined indicative for tumour cell apoptosis. As shown in Figure 8, mean caspase activity varied between 0.9 (August) and 2.2 (June), and 7 of the 12 sampling months (January, February, April, May, June, July, and December) were above the 1.5-fold threshold (higher *n*-fold caspase-3/-7 activity values indicate more apoptosis induction). One-way ANOVA testing indicated that, among the 12 sampling months, there was an overall significant effect of sampling month on *n*-fold caspase activity (*F*(11, 24) = 2.688, *p* = 0.02). Through the application of Tukey’s pairwise post-hoc testing, the data confirm that the *n*-fold caspase activity was significantly highest in June. Moreover, the most potent extracts exerted similar pro-apoptotic effects to the cytostatic drug gemcitabine (2.1-fold caspase-3/7 activity), which is used as standard chemotherapy in the treatment of pancreatic carcinoma patients. The other two cell cytotoxicity assays did not produce any mean inhibition levels above the 20% threshold for the A-549 lung carcinoma cells and MB-231 breast cancer cells (data not shown). No activity was observed against HaCaT cells, implying the lack of general toxicity of the crude organic extracts (data not shown).

## 3. Discussion

### 3.1. Optimisation of a PLE Protocol for F. vesiculosus

Among the parameters affecting the PLE process, the nature of the solvent, the temperature, and the extraction time represent the most critical. While pressure usually has a low influence on the result, temperature often increases extraction yields [41]. The yield is, however, not a sufficient indicator of chemical diversity, and higher temperature can be detrimental to the extraction of specific chemical classes, such as carotenoids [42]. Our PLE protocol intended to extract the largest part of the metabolome through a successive extraction performed with solvents of different polarities, namely MeOH and DCM, consecutively to a water rinse step aiming to remove salts and polar primary metabolites.

Three-level definitive screening designs highlighted the effectiveness of higher operating temperatures for increasing yield from both water and organic solvent extractions. In the case of the water extraction phase, three extraction cycles further increased the extract yield. This may be due to the increased solubility of high-molecular-weight (HMW) phlorotannins and fucoidans with elevated temperatures. Such molecules would greatly increase total yield, though not be detectable given the experimental mass detection limits of the UPLC-MS equipment used herein. Despite the elevated yield, higher temperatures were not found to significantly increase or decrease the chemical diversity used in this study; the number and area of UV and ELSD peaks, and the number of molecular networking nodes and clusters. Increasing the yield of HMW polymeric compounds was not a priority for this study, as further analysis is limited. Besides temperature, there was also no significant impact of the number or length of organic solvent static cycles on the various measures of chemical diversity. High extraction temperatures (50–200 °C) in PLE were previously reported to be detrimental to total phenolic yield and antioxidant activity of macroalgal extracts compared to traditional solid–liquid extraction (SLE) performed at ambient temperature and pressure [15]. This could not be quantified in this study due to the focus on the general metabolite profile and not a specific quantification of phenolic content.

Current literature detailing PLE parameters for *F. vesiculosus* and other brown algae focuses on the optimisation of individual compounds or specific chemical classes [43,44] rather than the whole detectable metabolome as targeted in the present study. For instance, PLE extraction conditions for maximal fucoidan yield in *Saccharina japonica* were found to be 140 °C and 50 bar, and used an aqueous solution of 0.1% NaOH [45]; however, in this case, the optimisation was not performed using a statistical DoE. In the present study, temperature was experimentally limited to 40 °C, the pressure was set to 100 bar (preset by the ASE system), and the extraction solvents were, respectively, MeOH and DCM, preceded by a water rinse phase. Although fucoidans were not identified in our study due to their larger molecular mass, which exceeds the capacity of the UPLC-MS equipment used, we presume that they were partly extracted during the initial water extraction. Suggested optimal conditions for the extraction of algal-derived fucoxanthin available in the literature vary. A PLE method using ethanol injected at high temperature (110 °C) and pressure (>100 bar) was used by Shang et al. [17], who also combined a statistical DoE approach with PLE to extract the brown alga *Eisenia bicyclis*. The Plackett–Burman DoE used in this case is a two-level factor design and requires more experimental runs than the DSD used in the present study; additionally, the study only targeted fucoxanthin. Using a non-statistical DoE approach in tandem with a basic semi-continuous flow system, Goto et al. [46] suggested an optimal extraction pressure of 5.9 bar when using liquefied dimethyl ether at ambient temperatures to extract carotenoids and lipids from the brown alga *Undaria pinnatifida*. Despite the contrasting extraction approaches, both studies extracted fucoxanthin with equal yield: 0.4 μg/mg. As for the long-chain fatty acid content of *F. vesiculosus* extracts, PLE conditions were reported to be optimal using ethyl acetate as a solvent when operating at 120 °C and 100 bar for 10 min [18]; however, this study did not use a statistical DoE. Although the processes were optimised for specific compounds rather than extracting the whole metabolome, similar results derived from two distinct methods indicate the flexibility of PLE and the need for a solvent-specific optimisation step.

The optimisation of the water extraction step produced similar results from the two DSD sequence repeats. Temperature was shown to significantly increase extraction yield in both cases. A comparison of the two DSD sequence repeats of the organic solvent extraction step indicates that the only significant experimental variable was the extraction temperature, which in both cases was found to increase the extraction yield. As there was no overall significant effect of the experimental variables on the measures of chemical diversity in the organic extracts, all such variables could be set to their most time- and solvent-efficient levels without compromising measurable chemical diversity. Despite the established body of literature in which DoE approaches have been incorporated into PLE to create optimal processes [47,48], the statistical designs employed in these studies generally require more experimental runs than DSDs. A similar study by Ain et al. [49] used a five-level response surface methodology (RSM) design to optimise the yield of oleoresin from lemongrass with PLE. Whilst there were only three independent variables in this design (temperature, pressure, and static time), the five-level design of the RSM required a total of 20 experimental runs. Comparably, the DSD used in the present study optimised six independent variables with only a 13-run experiment. Lu et al. [50] optimised the extraction of carotenoids from another brown alga *Laminaria japonica* using liquid extraction with a similar though less-sophisticated RSM design. The reduction of sample material required by combining PLE and DSDs is of particular interest to natural product chemistry whereby supply is often a limiting factor.

### 3.2. In-Depth Untargeted Metabolomics of F. vesiculosus Extracts

As there was no known previous work establishing a general metabolome analysis for *F. vesiculosus*, all monthly extracts were analysed by employing a comparative untargeted metabolomics approach to identify consistent UPLC-MS chromatogram features indicating universal major compounds. Sixteen distinct recurring peaks, consisting of 20 compounds, could be identified among all monthly chromatograms. Dereplication work by a molecular network and manual annotations by using multiple public and commercial databases allowed for putative identification hits for 44 recurring compounds in the *F. vesiculosus* metabolome, including those visibly identifiable on the monthly UPLC-MS chromatograms. These 44 compounds fell into six different chemical groups: phlorotannins, carotenoids, chlorophylls, betaine lipids, phospholipids, and tocopherols.

Despite being one of the best-studied compound groups in *F. vesiculosus*, only one representative of the phlorotannins was identified in the samples. A comparison of the molecular weight and fragmentation patterns with the literature indicates that the compound is a tetramer phlorotannin, potentially a fucophloroethol, due to their described prevalence in *F. vesiculosus* [34]. This classification is speculative. Tetrafucols or tetraphlorethols are other forms of phlorotannin tetramer also present in *F. vesiculosus*. Hermund et al. [34] also identified 12 other phlorotannin isomers from an Icelandic sample of *F. vesiculosus*, none of which matched compounds within the present study. Similarly, phlorotannin content from *F. vesiculosus* in other studies has been found to be extremely rich with an abundance of low-molecular-weight (*m*/*z* < 1000) structural isomers [51]. The discrepancy between the findings of the present study and those described in the literature may be due to geographical/seasonal variations of the samples used, plus the extraction solvent or high abundance of other compounds reducing the relative abundance of phlorotannins. As the extracts were produced from aggregated whole individuals of *F. vesiculosus*, each sample consisted of a complex mixture of biological matrices. The origin of these matrices may present a possible source of variance between samples, which was not accounted for. Although the aim of the study was to investigate the metabolome, it is possible that some replicates or samples from different months consisted of differing biological matrix origins, which in turn may have affected outcomes. Variance in metabolic production could also be affected by the ontogenetic stage of *F. vesiculosus*; however, care was taken to sample only adult individuals, so the only likely ontogenetic variance would be due to the seasonal spawning of the adults. Ethyl acetate has been reported to be particularly effective at extracting enriched amounts of phlorotannins from *F. vesiculosus* [13]. Since this study did not follow a targeted metabolome approach and aimed to describe the overall metabolite profile, EtOAc was not considered as an optional solvent. Methanol and chlorinated solvents (such as DCM) and their mixtures are generally the solvents of choice for exhaustive extraction of seaweeds for screening or bioactive compound isolation studies. Sathya et al. isolated an abundance of phlorotannins from *Cystoseira trinodis* (Fucaceae) using MeOH, which supports the choice of solvent used in the present study. The same study also utilised fractionation, as do many studies targeting phlorotannins from brown algae [10,52]. Fractionation of the monthly *F. vesiculosus* samples may provide a clarified view of the phlorotannins extracted using the PLE protocol in this study.

Sulphated polysaccharides were not identified in the sample extracts. Since fucoidan is a complex mixture of sulphated polysaccharides, they are commonly of a very high weight (6000 – >600,000 Da); as such, these are beyond the detection limit of the equipment (*m*/*z* 1200) used in this study. Although fucoidan is soluble in water at room temperature and may have been removed during the water rinse phase, extractions usually require the use of an organic solvent, such as ethanol, to break down the cell walls and liberate the polysaccharides [53]. This study, however, lacks metabolomics data for highly polar compounds, as the water extracts collected in the initial washing phase were not analysed. Cell walls of the brown algae are also well-described for their HMW phlorotannin content. Boettcher and Targett [54] profiled the phlorotannin size distribution of multiple *Fucus* species, including *F. vesiculosus*, finding that over 70% of phlorotannins present are over 10 kDa in size; well beyond the detection limits of the present study. These authors used 70% aqueous MeOH to extract the polyphenols, compared to the 100% MeOH used in the present study. Fractionation and further targeting of phenolic compounds in the monthly samples may highlight the presence of HMW phlorotannins. If significant levels of undetectable HMW phlorotannins were present in the samples, the amount of detectable low-molecular-weight phlorotannins may be proportionally decreased. Additionally, the more exhaustive extraction of other chemical groups, such as lipids, may proportionally decrease or mask the presence of phlorotannins.

Carotenoids are another major chemical class of interest found in brown algae, in particular fucoxanthin for its broad range of bioactivities [24]. Carotenoids were well-represented in the monthly *F. vesiculosus* samples. Fucoxanthin content in *F. vesiculosus* has previously been determined to account for 70% of the total xanthophyll content [55], which is similar to the present study, whereby the other xanthophylls dereplicated in the extracts (violaxanthin, neoxanthin, and zeaxanthin) exhibited much smaller peaks and TICs. The xanthophyll content in *F. vesiculosus* has previously been found to be higher in samples taken from a deeper (−4 m) location [19]. Samples in the present study were always collected from shallow depths (approximately −1 m) due to physical sampling constraints. It is possible that seasonal variations in the water depth had an additional effect on the xanthophyll content. Although xanthophylls are well-described for *Fucus* spp., other terpene classes, such as linear or cyclic diterpenes, sesquiterpenes, and meroterpenes that are common in many brown algal genera (e.g., *Bifurcaria*, *Sargassum*, *Dictyota* [56]), are not so well-described in *Fucus* spp. The dereplication analysis conducted in the present study, however, did not allow for the identification of such metabolites.

Lipids, specifically the polar, amphiphilic lipids, such as betaine lipids and phospholipids, represented the most diverse class of compounds in the monthly *F. vesiculosus* extracts. The molecular network analysis of the organic extracts identified five discrete lipid clusters: three betaine lipid clusters, two phospholipid clusters, and one large betaine lipid and phospholipid cluster. The clustering of betaine lipids with phospholipids is likely due to common fragmentation patterns between betaine lipids and phosphatidylcholines. The number of nodes putatively identified as betaine lipids were much greater than those of the phospholipids, indicating a general dominance of betaine lipids in the *F. vesiculosus* lipid profile. There were also comparably more lipid nodes than other major chemical classes, such as chlorophylls and carotenoids. The lipid composition of brown algae is well-documented in both content and diversity. Total lipid content in *Fucus* spp. generally varies between 2 and 4% of total dry mass [57,58]. A number of lipid classes of interest have been found in *Fucus* spp., particularly sterols and halogenated fatty acids [59,60]. Betaine lipids, of which many putative hits were identified in the monthly extracts, are a highly typical variety of lipids present in brown algae [61], which, along with phospholipids, are the major components of algal cell membranes [62]. While there are three possible forms of betaine lipids, the exact isomeric structure of the betaine lipids putatively identified in the samples could not be distinguished because of their common mass fragmentation patterns. Brown algae preferentially produce DGTA betaine lipids [63], which suggests that these are the isomeric forms of the present betaine lipids. The betaine lipids in brown algal species have been proposed as chemotaxonomic markers when other distinguishing methods are not available or are unsuccessful [64]. Whilst not as dominant as betaine lipids, 10 phospholipids were also putatively identified in the *F. vesiculosus* extracts. Of these, eight were identified as phosphatidylcholines, two were identified as phosphatidyethylamines, and the remaining two could not be classified further than phospholipids. Jones and Harwood [65] also noted the presence of phosphatidylcholines and phosphatidyethylamines in *F. vesiculosus*; however, in their study, the dominant phospholipid group was phosphatidyethylamines, which accounted for <10% of the total acyl lipids. It is difficult to compare these results to the present study, as only the number of compounds was recorded.

Although only represented in the monthly samples by α-tocopherol, marine macroalgae, including brown algae, have been described in the literature as sources of tocopherols, commonly known as dietary vitamin E [66]. The presence of dietary vitamin E in *F. vesiculosus* further reinforces its credentials as an edible superfood and/or food supplement. Whilst observably rich in important dietary compounds, such as tocopherols, carotenoids, and phlorotannins, the high levels of heavy metals (e.g., arsenic, cadmium, and mercury) and iodine in brown algae may present an obstacle to high-level commercial success as a health food [67].

### 3.3. Seasonal Changes in the Overall Metabolic Profile of F. vesiculosus

The analysis of the monthly changes in TIC of the major compounds in the monthly *F. vesiculosus* organic extracts presented general patterns of seasonality. These patterns generally correlated with the four principal chemical classes present in the extracts: chlorophylls, phlorotannins, lipids (two patterns), and carotenoids. Chlorophylls and their derivatives seemed to display an annual cycle of peaking during the winter months and dropping significantly during the summer. Ruokolahti and Rönnberg [68] observed similar patterns of low summer and high autumn and winter chlorophyll *a* content in *F. vesiculosus* from the northern Baltic Sea. The cause of the increase in algal chlorophyll *a* content in the winter was linked to the increased availability of dissolved nutrients during these months. A recent study on *F. vesiculosus* from the Kiel Fjord [69] reported increased chlorophyll levels in winter as a compensatory response to reduced light levels. Carotenoid pigment TIC followed a similar trend to the chlorophyll pigments; however, carotenoids were at their lowest around May as compared to the chlorophylls dropping during the mid-summer. Nomura et al. [70] measured the content of the photosynthetic carotenoid fucoxanthin [71] in two different brown algal species over the course of a whole year, and observed a similar pattern; fucoxanthin content was lowest in summer, but increased gradually in autumn before peaking in January. The same study observed that the general lipid content also peaked in winter. This is comparable to the present study; however, instead of a winter peak, the TIC of the identified lipids in *F. vesiculosus* increased towards the end of winter, peaking in March. This could be due to species-dependency and/or geographical variations, as Nomura et al. sampled *Sargassum horner* and *Cystoseira hakodatensis* from northern Japan. The general pattern of an increase in lipids in the colder months followed by a decrease in the warm summer months corresponds to findings in the literature [72,73]. The available literature on the seasonal variation of betaine lipids in algae is conflicting, whilst no study has been carried out on the seasonal variation of betaine lipid content in *Fucus* spp. *Ascophyllum nodosum* was found to have a high lipid content in May [74], which is similar to the findings of the present study. On the contrary, Blunden et al. [75] did not observe any seasonal variation in *A. nodosum* betaine content. Both studies noted a high degree of geographic variation in betaine lipid contents.

The phlorotannin tetramer TIC was lowest in winter and highest in the summer months. This is in contrast to the other three main chemical classes that generally exhibited higher TICs in the colder autumn/winter/spring months. Seasonal patterns found in the phlorotannin literature are similar, with the peak season generally falling between late spring and summer. Parys et al. [76] measured peak phenolic levels in *Ascophyllum nodosum* during July, whereas Connan et al. [77] recorded peak phlorotannin levels during the summer months in seven fucoid species, of which *F. vesiculosus* presented the highest phlorotannin levels. Variability in recorded phlorotannin maxima has been attributed to two possible factors, light availability and genetic variation. Although the production of phlorotannins was initially thought to be principally a plastic response to UV [21] due to their UV-absorbing properties, Jormalainen et al. [78] demonstrated that genetic variation in *F. vesiculosus* was more important than plastic responses to light exposure for the production of phlorotannins. Genetic variation was responsible for approximately 65% of observed variations in phlorotannin production, compared to only ~7% caused by phenotypic plasticity as a response to environmental manipulation, including grazing, nutrient availability, and light. In a subsequent study, Jormalainen and Honkanen [79] noted a strong degree of genetic variation in phenolic content in *F. vesiculosus* individuals depending on past environmental conditions. The production of the single phlorotannin tetramer detected in the present study is not conclusive evidence of a May peak in the sampled *F. vesiculosus*; so, a targeted approach to study polyphenolic compounds is necessary to establish a seasonal pattern. The 23 unidentified compounds that exhibited significant monthly variations in TIC may be significant components of the *F. vesiculosus* metabolome, and 13 of the 23 had a maximum TIC of over 1,000,000. The potential of these compounds to represent new molecules should be studied in future work.

### 3.4. Seasonal Changes in the Bioactivity of F. vesiculosus Extracts

Although no strong inhibition of the A-549 lung or MB-231 breast cancer cell lines by the monthly *F. vesiculosus* extracts was observed, significant inhibition of the pancreatic cancer cell line Panc1 was evident as indicated by the caspase-3/7 induction potential of the extracts. The published literature generally attributes the antitumour activity of *F. vesiculosus* to phlorotannins [36], fucoidan [80], and fucoxanthin [81]. Recorded caspase-3/7 induction potential values in this study were generally higher in the first half of the year (January, February) and late spring/early summer (May, June, July), compared to late summer and early autumn (August, September, October). The phlorotannin content of the monthly *F. vesiculosus* extracts appears to be the major identifiable Panc1 inhibition agent rather than carotenoid content given the observed pattern of caspase-3/7-induced cell apoptosis. The late spring/early summer phlorotannin tetramer peak mirrors the high Panc1 inhibition potential of the extracts during May, June, and July. Similarly, when the phlorotannin TIC declined between August and December, caspase-3/7 activity was also suppressed. Geisen et al. [35] observed the inhibition of various pancreatic cancer cell lines, including Panc1, by purified acetone extracts of *F. vesiculosus*; however, instead of measuring cell apoptosis, the study recorded cancer cell numbers as a measure of viability. It was noted that the mechanism of action of the *F. vesiculosus* extract was the inhibition of the cell cycle; similar to gemcitabine, the positive control used in the present study. A follow-up study found that the *F. vesiculosus* fractions with the highest activity against pancreatic cell lines had a very high polyphenolic content, indicating phlorotannins to be the key inhibitory compounds [36], which may support the findings of the present study. The general fluctuation in TIC of the carotenoids indicates the lowest carotenoid content during the spring months when caspase-3/7 activity was highest, whilst the highest TIC values appear to be during autumn, whereby caspase-3/7 activity was at its lowest during this time.

Free radical scavenging activity by the monthly extracts as measured by the DPPH assay indicated that the late spring and summer months (April, May, July, August) produced the highest DPPH free radical scavenging activity, whilst the lowest activity was observed in the colder spring and winter months (January, March, September, November). This pattern is similar to the observed fluctuation in the phlorotannin tetramer TIC, which peaked in late spring (May), and recorded its lowest values in January, March, and December. Although the recorded phlorotannin TIC does not fit exactly to the observed DPPH radical scavenging activity profile (high August activity, low August phlorotannin TIC), the phlorotannin tetramer was the only phlorotannin detected in the samples. Therefore, it may be that other summer-peaking phlorotannins that were not detected by the UPLC-MS are responsible for high DPPH free radical scavenging activity, as phlorotannins are well-described antioxidants [13,34]. It could also be that, while the high antioxidant activity in spring is caused by the presence of phlorotannins, the August peak may be a complementary effect caused by (an)other compound(s), e.g., carotenoids, with well-known antioxidative activity. Of the other significantly variable compounds present in the extracts, very few have high TIC in August. One carotenoid exhibited high TIC in August; however, the same compound also exhibited high TIC in the winter months when antioxidant activity was low. High summer and low winter TIC was observed in two betaine lipids. This may be coincidental, however, as no published literature could be found describing antioxidant or DPPH free radical scavenging activity by betaine lipids. The potential effect of carotenoids present in the extracts in the DPPH assay was considered; however, the crude extracts did not show any significant absorption at the test wavelength (517 nm) and concentration (100 μg/mL).

The antimicrobial assays conducted against the ESKAPE panel composed of six drug-resistant human pathogenic bacteria, two human fungal pathogens, and a small panel of algal and environmental pathogens, revealed that monthly *F. vesiculosus* extracts inhibited only the MRSA strain above the 20% threshold at the 100 μg/mL test concentration. A statistical analysis of the variation in MRSA inhibition by the monthly extracts indicated that the inhibition potential of the September replicates was significantly lower than that of the January and March replicates; however, a general seasonal trend could not be observed. One possible explanation for the extremely low September values in comparison to the rest of the year is the occurrence of a large storm on 14 September 2017 [82], one week before our sample collection (21 September 2017). This caused a 1-m drop in the water level below the mean, which may have uncovered algae in the sampling site. Otero et al. [18] showed that higher concentrations (2.5 mg/mL) of PLE ethanolic extracts of *F. vesiculosus* significantly inhibit *S. aureus* (49.8%) as well as the Gram-negative bacterium *E. coli* (30.7%). The gram-negative *E. coli* was not inhibited in the present study, albeit at a much lower test concentration. The same study also noted the relatively higher activity in *F. vesiculosus* extracts compared to other brown and green macroalgae. The antimicrobial effect of brown algae, particularly against Gram-positive bacteria, such as MRSA, has been well-documented in the literature. The source of anti-MRSA activity in brown algae is highly debated, with multiple possible contributory compounds; namely, phytosterols [83], phlorotannins [84], and sulphated polysaccharides [85]. Of the three chemical classes, the current study putatively identified only one phlorotannin in the monthly extracts. The high levels of MRSA inhibition could indicate high levels of undetected or unidentified phytosterol and/or fucoidan present in the *F. vesiculosus* extracts. Whilst there is an apparent seasonality in the inhibition of MRSA by the *F. vesiculosus* extracts, the observed pattern does not clearly correspond to either the observed chemical class seasonality patterns in the present study, or the debated MRSA-inhibiting compounds described in the literature. The abundance of the phlorotannin tetramer during January–March was comparable to that of September, when the greatest difference in anti-MRSA activity was observed. It is possible that multiple chemical classes inhibit MRSA; however, these compounds may have contrasting seasonality patterns, which would produce an underlying MRSA inhibition across all monthly samples. One option would be to test fractions, rather than the crude extracts, obtained from the monthly *F. vesiculosus* extracts against the MRSA, DPPH, and Panc1 assays. Such an approach combined with a more targeted metabolome analysis would be helpful, and this will be the subject of our future studies.

Although *F. vesiculosus* extracts produce evident MRSA growth inhibitory, radical scavenging, and pro-apoptotic activities, this study highlights the significant effect of the sampling month on these activities. The likely candidate agents for both activities are proposed to be phlorotannin. These have previously been reported to produce such activities; however, this study is the first one to establish a connection between the seasonal variations in these bioactivities and the seasonal fluctuations in phlorotannins. Thus, the importance of the sampling season/month to affect the chemical composition of biological samples is evident. This fact should be regarded in future sampling campaigns, especially for targeted chemistry and/or bioactivity studies, for chemical isolation, metabolomics, or other interests. Such an approach can maximize the yield of specific bioactive compounds for in-depth studies.

## 4. Materials and Methods

### 4.1. Algal Material

Three replicates of *F. vesiculosus* were collected every month for an entire calendar year (January–December 2017) from the same shallow (approx. −1 m) littoral patch at Bülk, outer Kiel Fjord, in the mouth of the Baltic Sea (54°27′15.3″ N, 10°11′56.1″ E). Replicates consisted of a random selection of adult *F. vesiculosus* individuals within the collection area. The algal samples were stored in sealed plastic bags (containing seawater) in chilled boxes and transported to the laboratory immediately after collection. Algal material was then sorted and rinsed with deionised water to remove excess salt, macroorganisms and epiphytes. Heavily fouled individuals were discarded; individuals presenting minor fouling were carefully cleaned by hand to remove epibionts. The algal material was freeze-dried and ground to a semi-coarse powder using a Pulverisette 14 (1.0 mm sieve ring, trapezoidal perforation, 12 ribs rotor; Fritsch GmbH, Idar-Oberstein, Germany).

### 4.2. Pressurised Liquid Extraction

PLE [86] was performed using an ASE 350™ (Dionex, Thermo Fisher Scientific, Sunnyvale, CA, USA) to rapidly extract all monthly samples. Thirty-four millilitres (34 mL) ASE 350™ cells were filled with a mixture of 0.5 g dried algal powder and 10 g acid-washed sand (Grüssing GmbH, Filsum, Germany) sandwiched between two packing layers of pure acid-washed sand. Two cellulose filters (Φ 30 mm for ASE™; Dionex Thermo Fisher Scientific, Sunnyvale, CA, USA) were placed at the bottom of the extraction cell, and one cellulose filter was placed above the top layer of packing sand. All extractions consisted of an initial water-rinsing phase mainly to remove water-soluble salts from the material; this was followed with an organic solvent extraction sequence, firstly with MeOH (MS-grade; AppliChem GmbH, Darmstadt, Germany) and subsequently with DCM (>99.8%; AppliChem GmbH, Darmstadt, Germany) to create a 1:1 crude extract mixture of the two solvents. Extraction sequences and the ASE 350™ setup were programmed using Chromeleon 7 (Dionex, Thermo Fisher Scientific, Sunnyvale, CA, USA) software installed on a secondary computer connected to the ASE device. The purge time for all extractions was set to 100 s. The rinse volume was set to 30% of the total cell volume (10.2 mL). The water extraction phase consisted of three 10 min static cycles. Each of the MeOH and DCM extraction phases consisted of one 5 min static cycle. In addition to the 3 replicates from each of the 12 sampling months (36 in total), three extraction blanks were also produced by extracting cells filled with only acid-washed sand. The combined MeOH and DCM organic solvent extracts were subsequently dried using a Syncore Polyvap R-12 parallel evaporator (Büchi GmbH, Flawil, Switzerland). Dried extracts were then dissolved in MeOH (absolute ULC/MS-grade; Biosolve Chimie SARL, Dieuze, France) and filtered through 0.2 μm polytetrafluoroethylene pre-filters (VWR International GmbH, Darmstadt, Germany) to remove particles. Extracts were dried and stored in the dark at 5 °C until chemical analyses.

### 4.3. Determination of Optimal Extraction Conditions

#### 4.3.1. Definitive Screening Design

For each optimization extraction, the following parameters were universal: 1 pre-extraction rinse (10 mL of extraction solvent), ‘standard’ extraction mode, 100 s purge time, and 1500 psi pressure. DSD tables were generated using JMP 14 statistical software (SAS Institute, Cary, NC, USA). All DSD tables used a three-level design: for each input variable, there was a low, medium, and high factor level. DSD matrices were then generated with a permutation of factor levels for each input variable to enable an analysis of the factor–variable relationship with 2*n* + 1 runs. JMP 14 uses a root-mean-square analysis of input variable regression models to identify statistically significant main effect variables.

#### 4.3.2. The Water Extraction Step

The DSD table generated for the optimisation of the water extraction step consisted of 13 runs with six input variables. The input variables were temperature, sample weight, ratio of sand:*Fucus* material, number of static cycles (injections into cell), static time (time in cell), and rinse volume (amount of solvent released into collection vial as a percentage of cell volume). DSD runs were ordered by ascending temperature for practical reasons (Table 1).

Each DSD run was extracted in succession over the course of one night. Two repeats of the 13-run DSD table were performed to validate obtained water extraction parameter results. Both repeats used *F. vesiculosus* material sampled from March 2017. The output variable (measure of success) for the optimisation of the water extraction step was the extraction yield, calculated as:

Yield (%) = [dried extract mass (g) / mass of dried *F. vesiculosus* (g)] × 100

#### 4.3.3. The Organic Solvent Extraction Step

The organic solvent extraction step with MeOH and DCM was performed using a subsequent three-level DSD table (Table 2), which consisted of 13 runs with five input variables. For each run in the DSD table, the algal material was first extracted with the optimised water extraction protocol, followed by the MeOH step, and finally the DCM step. Once each run was fully extracted, the next commenced immediately. The five input variables were temperature, number of MeOH static cycles (MeOH injections into cell), MeOH static time (MeOH time in cell), number of DCM static cycles (DCM injections into cell), and DCM static time (DCM time in cell).

Each DSD run was extracted in succession over the course of one day/night period. Two repeats of the 13-run DSD table were performed to validate the results of the organic solvent extraction parameters. One repeat used *F. vesiculosus* material sampled from February 2017, and the other from June 2017, to account for possible seasonal effects.

#### 4.3.4. Determination of the Optimum Extraction Conditions and Analysis of Monthly Extracts

The aim of the organic solvent optimisation step was to maximize both the chemical diversity and yield of the organic solvent extractions. The following output variables were used to measure the relative effect of each input variable on the chemical diversity of the organic solvent extracts: number of GNPS molecular networking nodes, number of GNPS molecular networking clusters, number of chromatogram peaks (ELSD signal, 280 nm UV, 405 nm UV), and area of chromatogram peaks (ELSD signal, 280 nm UV, 405 nm UV). These optimisation variables were collected using HPLC-PDA-ELSD and HPLC-MS^2^ (in tandem with molecular networking).

##### HPLC-PDA-ELSD

ELSD signal and UV absorption variables were measured by a VWR Hitachi Chromaster HPLC system (VWR International GmbH, Darmstadt, Germany) coupled with a PDA detector 5430 (VWR International GmbH, Darmstadt, Germany) and an ELSD 90 LT detector (VWR International GmbH, Darmstadt, Germany). Extracts were dissolved in MeOH at a concentration of 5 mg/mL. Ten microlitres (10 μL) of dissolved extract solution was then injected at 10 °C into an Onyx monolithic C18 column (100 × 30 mm) operating at 40 °C at a flow rate of 1 mL/min. The elution gradient started at 1% B (ACN + 0.1% HCOOH), and changed to 75% B within 2 min, followed by 95% B within 22 min and then 100% at 23 min at which it stayed for 7 min. The gradient then returned to original 1% B conditions until 35 min. The PDA detector recorded UV absorption between 200 and 600 nm. The ELSD signal was recorded by the ELSD detector set at 40 °C, with a gain of 8 and a filter of 2 s.

##### UPLC-PDA-ESI-QTOF-MS^n^

UPLC-PDA-MS was performed using an Acquity I-Class UPLC system (Waters, Milford, MA, USA) connected to an Acquity PDA eλ detector and a Xevo GS-XS QTof mass spectrometer (Waters, Milford, MA, USA). Extracts were dissolved in MeOH at a concentration of 1 mg/mL. An amount of 0.7 μL of dissolved extract solution was then injected at 10 °C into a 2.1 × 100 mm UPLC HSS T3 column (Waters, Milford, MA, USA) operating at 40 °C at a flow rate of 0.6 mL/min. The elution solvents used were A: H_2_O (absolute ULC/MS-grade; Biosolve Chimie SARL, Dieuze, France) + 0.1% HCOOH (ULC/MS optigrade; LGC Standards, Teddington, UK); B: ACN (absolute ULC/MS-grade; Biosolve Chimie SARL, Dieuze, France) + 0.1% HCOOH. The elution gradient started at 1% B and changed to 40% B within 2.5 min, between 2.5 min and 13.5 min B increased from 40% to 100%, which it stayed at for 1 min (14.5 min total time). The gradient then immediately returned from 100% B to the original 1% B conditions, at which it stayed from 14.6 min until the end of the elution at 18 min. Data were collected in both the MS and data-dependent acquisition (DDA) modes. MS and MS/MS fragmentation spectra were recorded in positive mode using the following parameters: desolvation temperature: 550 °C, desolvation gas flow: 1200 L/h, cone gas flow: 50 L/h, cone voltage: 40 V, capillary voltage: 0.8 kV, collision energy ramp: 6-60/9-80 (low mass/high mass), source temperature: 150 °C, acquisition range 50–1600 *m*/*z*, scan times: 0.1. UV absorption spectra were recorded between 190 and 600 nm. MassLynx (Waters, Milford, MA, USA) was used for the data acquisition. 

##### Molecular Networking

Molecular networking was performed on the UPLC-MS^2^ data using the same parameters applied during the organic solvent optimisation step of the experiment. Clusters containing multiple nodes with spectra matching GNPS library spectra were putatively assigned the chemical classes of the matched compounds to assist general dereplication. Visible UPLC-MS chromatographic peaks recurring in every sampling month were manually identified to establish those that are consistently present in the whole sampling year in order to form a general metabolic profile for *F. vesiculosus*. The dereplication of compounds only present in the algal extracts (i.e., absent in blank extracts) was performed using *m*/*z* values of the parent ion in UPLC-MS chromatograms, MS fragmentation patterns, and UV absorption spectra. Putative hits were identified on the basis of GNPS spectral matches as well as manual searches using the key words “alga”, “brown alga”, and “Fucus” in the Dictionary of Natural Products on DVD (CRC Press, Taylor & Francis group, 2010), MarinLit (Royal Society of Chemistry, 2017), and the Universal Natural Products Database [87], respectively.

To apply statistical testing to identify significant differences between the monthly sample chromatograms, the UPLC-MS data recorded in MS mode were uploaded to the XCMS Online [40] metabolomics platform, which subsequently allowed for automatic peak alignment of the UPLC-MS and a statistical analysis of the compound intensities. Before uploading to the platform, the Waters Compression Tool (Waters, Milford, MA, USA) was used to reduce MS data noise and the file size. Replicates were arranged by month and submitted as a multi-group job to the platform using the Waters High-Res POS parameter with matchedWave feature detection. The statistical test applied to the data was a parametric analysis of variance (ANOVA). Peak alignment results were downloaded from XCMS Online and subjected to a series of filters to isolate major compounds in the extracts. Features were filtered to remove contaminant compounds (features with a control extract TIC of ≥5% maximum sample extract TIC), minor compounds (features with a maximum TIC and intensity ≤100,000), and isotopes (lower TIC isotopes were removed). These compounds were then further filtered by *p*-value to include only those that indicated a significant effect (*p* = < 0.05) of sampling month on peak intensity to identify the main seasonally variable compounds within the samples.

### 4.4. Bioassays

#### 4.4.1. Antimicrobial Activity

Crude extracts from all monthly replicates and extraction blanks were tested for bioactivity against multiple bacterial and fungal pathogen strains of interest to pharmaceutical and ecological science research. The antimicrobial strains and their strain-specific culture conditions are detailed in Table S3. Strains were prepared in their specific media and left overnight at 160 rpm (*Enterococcus faecium* without shaking), in strain-specific temperatures to culture, at which point they were diluted with medium to a strain-specific optical density. For all crude extracts and extraction blanks, a stock solution of 20 mg/mL in DMSO was prepared, diluted with medium to a final assay concentration of 100 μg/mL, and added to a 96-well microplate for each strain to be tested against. The wells were then inoculated with 200 μL of media-diluted test organisms and incubated at the strain-specific temperature and time and 200 rpm (*E. faecium* without shaking). To detect the inhibitory effect of the extracts, 10 µL of a resazurin solution (0.3 mg/mL phosphate-buffered saline) was added to the microplates (except *E. faecium* and *Cryptococcus neoformans*), incubated again for 5–30 min, and the fluorescence signal (560 nm/590 nm) was measured using the microplate reader (Tecan Infinite M200, Tecan, Crailsheim, Germany). For *E. faecium,* the pH indicator bromocresol purple was used to determine the acidification caused by growth. For *C. neoformans*, the absorbance at 600 nm was measured. The resulting values were compared with the positive control (chloramphenicol, 10 µM), a negative control (no compound), and a DMSO control on the same plate. A threshold of 20% was used to identify extracts causing inhibition.

#### 4.4.2. Free Radical Scavenging Capacity

The free radical scavenging activity of the crude *F. vesiculosus* extracts was evaluated using DPPH (Sigma-Aldrich, Munich, Germany). One hundred microlitres (100 μL) of algal extracts dissolved in MeOH were added to a 96-well microplate. A further 100 μL of 200 μM DPPH solution was added to each well. The microplate was incubated for 30 min in darkness at room temperature, after which the absorption at 517 nm was measured. The transition from a purple colour, caused by the DPPH radical, to a yellow colour caused by the reduction to DPPH-H (2,2-diphenyl-1-picrylhydrazin) indicates antioxidant activity in the test sample and is detected at an absorbance of 517 nm. The extract values were compared with a positive control (ascorbic acid, 100 µM) and a negative control (no test samples).

#### 4.4.3. Anticancer and Cytotoxic Activity

A-549 lung carcinoma cells, MDA-MB-231 breast cancer cells, and HaCaT human keratinocytes, all obtained from Cell Line Service (Eppelheim, Germany), were tested for assessing anticancer and cytotoxic activity. An amount of 1 × 10^4^ cells were seeded in each well of a 96-well microplate and cultivated for 24 h in RPMI medium supplemented with 10% fetal bovine serum, 100 U/mL penicillin, and 100 mg/mL streptomycin at 37 °C under a humidified atmosphere and 5% CO_2_. Afterwards, the medium was removed, and 100 μL of fresh medium containing the test extracts at a concentration of 100 μg/mL were added to the wells. Tamoxifen was used as the positive control, and wells without any test sample were used as negative control. Plates were then incubated for 24 h at 37 °C, and the CellTiter-Blue Cell Viability Assay (Promega, Mannheim, Germany) was performed according to the manufacturer’s instructions. After 2 h at 37 °C, the fluorescence was measured using the microplate reader Tecan Infinite M200 with excitation at 560 nm and an emission of 590 nm.

The impact of extracts on apoptotic behaviour of the human pancreatic carcinoma cell line Panc 1 (purchased from the American Type Culture Collection (ATCC)), which was sensitive to *F. vesiculosus* extract [30,31], was analysed by determination of caspase-3/7 activity using the Caspase-Glo^®^ 3/7 assay (Promega, Mannheim, Germany), according to the manufacturer’s instructions. For this purpose, 5 × 10^3^ cells/well were seeded in 100 µL RPMI medium (supplemented with 10% FCS, 1% l-Glutamine, and 1% sodium pyruvate) in a 96-well plate. After 24 h, cells were treated with 1 µl DMSO (control) or with extracts at a concentration of 100 μg/mL. Gemcitabine at 10 μg/mL was used as a positive control. All samples were measured in duplicates. Chemoluminescence signals were determined using an Infinite^®^ 200 PRO microplate reader (Tecan, Crailsheim, Germany). Values were normalised to DMSO-treated cells, and data are expressed as *n*-fold caspase-3/7 activity of DMSO-treated cells. A 1.5-fold caspase activity equated to a caspase activation of 50%, and was used as the threshold for significant inhibition by *F. vesiculosus* extracts.

## 5. Conclusions

In addition to establishing a PLE protocol for the extraction of the whole *Fucus vesiculosus* metabolome using a sophisticated design of experiment approach, the impact of sampling season on the bioactivity and metabolite profile was highlighted by the corresponding seasonal chemical profiles as well as anti-MRSA, DPPH free radical scavenging, and caspase-induced Panc1 cancer cell apoptosis.

## Figures and Tables

**Table 1:** Three-level DSD table for the optimisation of the water extraction step.

DSD Run	Temp. (°C)	Sample Weight (g)	Ratio Sand:*Fucus*	Static Time (min)	No. of Static Cycles	Rinse Volume (% of Cell)
1	20	0.5	10:1	11	2	120
2	20	0.5	20:1	8	3	30
3	20	1.0	10:1	5	1	120
4	20	1.5	20:1	5	3	75
5	20	1.5	10:1	11	1	30
6	40	0.5	15:1	5	1	30
7	40	1.0	10:1	8	2	75
8	40	1.5	15:1	11	3	120
9	60	0.5	20:1	5	3	120
10	60	0.5	20:1	11	1	75
11	60	1.0	15:1	11	3	30
12	60	1.5	20:1	8	1	120
13	60	1.5	10:1	5	2	30

**Table 2:** Three-level DSD table for the optimisation of the organic solvent extraction step.

Run	Temp. (°C)	MeOH Static Time (min)	No. of MeOH Static Cycles	DCM Static Time (min)	No. of DCM Static Cycles
1	20	5	1	11	2
2	20	5	3	8	3
3	20	8	3	5	1
4	20	11	1	5	3
5	20	11	2	11	1
6	40	5	1	5	1
7	40	8	2	8	2
8	40	11	3	11	3
9	60	5	2	5	3
10	60	5	3	11	1
11	60	8	1	11	3
12	60	11	1	8	1
13	60	11	3	5	2

## Supplementary Materials

The following materials are available at http://www.mdpi.com/1660-3397/16/12/503/s1. Table S1: Dereplication table of the 44 compounds identified in the *F. vesiculosus* extracts, Table S2: Table of the 54 compounds significantly varying with sampling month, Table S3: Pathogen strains and their specific culture conditions. Figure S1: Linear regressions of the relationships between the five MeOH:DCM extraction phase optimisation variables and the measures of chemical diversity, Figures S2–S8: Seasonality box plots for all compounds significantly varying with sampling month.
