# Supplementary material for: Seasonal Variations in the Metabolome and Bioactivity Profile of Fucus vesiculosus Extracted by an Optimised, Pressurised Liquid Extraction Protocol
Source: Mar Drugs. 2018 Dec 13;16(12):503. doi: 10.3390/md16120503 (PMC6315544; doi:10.3390/md16120503)
Supplement: Supplementary file 1 [file marinedrugs-16-00503-s001.pdf]

**Supplementary materials**

# Seasonal Variations in the Metabolome and Bioactivity Profile of *Fucus vesiculosus* Extracted by an Optimised, Pressurised Liquid Extraction Protocol

**Edwin Heavisides [1], Caroline Rouger [1,†], Anna F. Reichel [1], Cornelia Ulrich [1], Arlette Wenzel-Storjohann [1], Susanne Sebens [2] and Deniz Tasdemir [1,3,*]**

[1] GEOMAR Centre for Marine Biotechnology (GEOMAR-Biotech), Research Unit Marine Natural Products Chemistry, GEOMAR Helmholtz Centre for Ocean Research Kiel, Am Kiel-Kanal 44, 24106 Kiel, Germany; edheavisides@outlook.com (E.H.); caroline.rouger@u-bordeaux.fr (C.R.), reichel-anna@gmx.de (A.F.R.); cornelia.ulrich@mpimf-heidelberg.mpg.de (C.U.); awenzel-storjohann@geomar.de (A.W.-S.)

[2] Institute for Experimental Cancer Research, Christian-Albrechts-University Kiel (CAU) and University Medical Center Schleswig-Holstein (UKSH) Campus Kiel, Arnold-Heller-Str. 3, Building 17, 24105 Kiel, Germany; susanne.sebens@email.uni-kiel.de

[3] Faculty of Mathematics and Natural Sciences, Christian-Albrechts-University Kiel (CAU), Christian-Albrechts-Platz 4, 24118 Kiel, Germany

* Correspondence: dtasdemir@geomar.de; Tel.: +49-431-600-4430

† Current address: Univ. de Bordeaux, UFR des Sciences Pharmaceutiques, Unité de recherche Œnologie EA 4577, USC 1366 INRA, ISVV, 210 Chemin de Leysotte, CS 50008, 33882 Villenave d'Ornon, France.

| Table No. | Page No. |
|---|---|
| **Table S1.** Dereplication of compounds identified in monthly *F. vesiculosus* organic extracts from Jan. 2017 – Dec. 2017, based on UPLC-PDA-(ESI+)$MS^2$ data. | 3 |
| **Table S2.** Dereplication table of the 54 compounds significantly varying with collection month. | 6 |
| **Table S3.** Pathogen strains and their strain-specific culture conditions. | 13 |

| Figure No. | |
|---|---|
| **Figure S1.** Statistical linear regression analyses of the relationships between the five extraction variables and: (A) number of ELSD signal peaks (1st repeat); (B) average number of UV peaks (2nd repeat); (C) 405 nm UV peak area (2nd repeat); (D) number of 280 nm UV peaks (2nd repeat). | 14 |
| **Figure S2.** Seasonality box plots depicting monthly median (bold), 1st-3rd quartiles (lower-upper box limits), and minimum and maximum (lower and upper bars) total ion count for compounds I-VIII. | 15 |
| **Figure S3.** Seasonality box plots depicting monthly median (bold), 1st-3rd quartiles (lower-upper box limits), and minimum and maximum (lower and upper bars) total ion count for compounds IX-XVI. | 16 |
| **Figure S4.** Seasonality box plots depicting monthly median (bold), 1st-3rd quartiles (lower-upper box limits), and minimum and maximum (lower and upper bars) total ion count for compounds XVII-XXIV. | 17 |
| **Figure S5.** Seasonality box plots depicting monthly median (bold), 1st-3rd quartiles (lower-upper box limits), and minimum and maximum (lower and upper bars) total ion count for compounds XXV-XXXII. | 18 |
| **Figure S6.** Seasonality box plots depicting monthly median (bold), 1st-3rd quartiles (lower-upper box limits), and minimum and maximum (lower and upper bars) total ion count for compounds XXXIII-XL. | 19 |
| **Figure S7.** Seasonality box plots depicting monthly median (bold), 1st-3rd quartiles (lower-upper box limits), and minimum and maximum (lower and upper bars) total ion count for compounds XLI-XLVIII. | 20 |
| **Figure S8.** Seasonality box plots depicting monthly median (bold), 1st-3rd quartiles (lower-upper box limits), and minimum and maximum (lower and upper bars) total ion count for compounds XLIX-LIV. | 21 |

**Table S1.** Dereplication of compounds identified in monthly *F. vesiculosus* organic extracts from Jan. 2017 – Dec. 2017, based on UPLC-PDA-(ESI+)MS² data.

| Comp. ID | $t_R$ (min) | Chemical class | Putative identification | Mol. Formula (M) | MS¹ (*m/z*) | Error (Δppm) | Confidence level[a] | MS² (*m/z*) | UV $\lambda_{max}$ (nm)[b] |
|---|---|---|---|---|---|---|---|---|---|
| 1 | 0.98 | Phlorotannin | Fucodiphlorethol A, B, C, D, E, G or Difucophlorethol A or Tetrafucol A, B or Tetraphlorethol A, C, E | $C_{24}H_{18}O_{12}$ | 499.084 [M+H]⁺ | 7.3 | 2 | 481.09, 463.08, 411.08, 395.09, 393.07, 355.06, 337.05, 327.06, 287.07, 285.05, 271.04, 269.05 139.04 | |
| 2 | 6.12 | Phosphatidylcholine | LysoPC[c] 20:5 | $C_{28}H_{48}NO_7P$ | 542.321 [M+H]⁺ | 6.8 | 2 | 524.33, 184.08, 104.11 | |
| 3 | 6.27 | Betaine lipid | LysoDGTSA[d] 14:0 | $C_{24}H_{47}NO_6$ | 446.339 [M+H]⁺ | 20.5[f] | 2 | 428.35, 285.25, 236.16, 144.11 | |
| 4 | 6.29 | Betaine lipid | LysoDGTSA 18:3 | $C_{28}H_{49}NO_6$ | 496.350 [M+H]⁺ | 27.8[f] | 2 | 478.37, 236.15, 144.11 | |
| 5 | 6.31 | Betaine lipid | LysoDGTSA 20:5 | $C_{30}H_{49}NO_6$ | 520.354 [M+H]⁺ | 18.9[f] | 2 | 502.37, 236.15 | |
| 6 | 6.68 | Phosphatidylethanolamine | LysoPE[e] 20:4 | $C_{25}H_{43}NO_7P$ | 502.283 [M+H]⁺ | 20.6[f] | 2 | 422.16, 379.17, 361.28 | |
| 7 | 6.81 | Betaine lipid | LysoDGTSA 18:2 | $C_{28}H_{51}NO_6$ | 498.376 [M+H]⁺ | 6.9 | 2 | 480.37, 361.29, 236.16, 144.11 | |
| 8 | 6.88 | Betaine lipid | LysoDGTSA 20:4 | $C_{30}H_{51}NO_6$ | 522.377 [M+H]⁺ | 4.7 | 2 | 504.38, 236.16, 173.03 | |
| 9 | 7.27 | Betaine lipid | LysoDGTSA 20:3 | $C_{30}H_{53}NO_6$ | 546.369 [M+Na]⁺ | 14.7[f] | 2 | 528.39, 236.16 | |
| 10 | 7.45 | Betaine lipid | LysoDGTSA 16:0 | $C_{26}H_{51}NO_6$ | 474.373 [M+H]⁺ | 13.6[f] | 2 | 456.40, 351.14, 313.29, 236.16, 144.11 | |
| 11 | 7.52 | Betaine lipid | LysoDGTSA 18:1 | $C_{28}H_{53}NO_6$ | 500.392 [M+H]⁺ | 6.2 | 2 | 482.39, 422.15, 236.16 | |
| 12 | 7.76 | Carotenoid | Fucoxanthin dehydrated | $C_{42}H_{58}O_6$ | 641.427 [M+H-H₂O]⁺ | 10.0[f] | 2 | 549.37, 221.16, 203.15 | |
| 13 | 8.40 | Carotenoid | Violaxanthin or Neoxanthin | $C_{40}H_{56}O_4$ | 583.420 [M+H-H₂O]⁺ | 8.4 | 2 | 491.37, 283.22, 221.16, 165.10 | |
| 14 | 9.30 | Chlorophyll | Chlorophyll *c*2 | $C_{35}H_{28}MgN_4O_5$ | 609.204 [M+H]⁺ | 8.5 | 2 | 591.23, 549.21, 534.23, 532.19 | |
| 15 | 9.61 | Chlorophyll | | | 623.413 [M+H]⁺ | | 3 | 605.42, 587.42, 549.34, 531.34, 295.17, 259.13 | 224, 450 |
| 16 | 10.71 | Phosphatidylcholine | | | 782.590 [M+H]⁺ | | 3 | 750.57, 572.39, 554.39, 446.37, 428.35, 184.08 | |
| 17 | 10.71 | Chlorophyll | Pheophytin *b* | $C_{55}H_{72}N_4O_6$ | 885.602 [M+H]⁺ | 55.3[f] | 2 | 607.30, 547.28 | |
| 18 | 10.93 | Carotenoid | Fucoxanthinol | $C_{40}H_{56}O_5$ | 599.408 [M+H-H₂O]⁺ | 3.4 | 2 | 581.42, 411.28, 355.25, 277.21, 263.19, 213.13, 199.15, 185.14, 173.14, 127.11, 109.10 | 224, 265, 330, 447, 470 |
| 19 | 10.94 | Carotenoid | Fucoxanthin | $C_{42}H_{58}O_6$ | 681.421 [M+Na]⁺<br>659.435 [M+H]⁺ | 11.6[f]<br>6.4 | 1[g]<br>1 | 663.42, 621.41, 603.40, 527.33<br>641.46, 581.43, 109.11 | 224, 265, 330, 447, 470 |
| 20 | 11.20 | Chlorophyll | Pheophorbide *a* | $C_{35}H_{36}N_4O_5$ | 593.279 [M+H]⁺ | 4.4 | 2 | 533.27 | 224, 265, 330, 409, 505, 535 |
| 21 | 11.35 | Phosphatidylcholine | | | 810.625 [M+H]⁺ | | 3 | 778.60, 313.28, 184.08 | |
| 22 | 11.40 | Betaine lipid | DGTSA 32:4 | $C_{42}H_{73}NO_7$ | 704.553 [M+H]⁺ | 9.2 | 2 | 494.37, 476.35, 446.38, 428.35, | |

| | | | | | | | | | |
|---|---|---|---|---|---|---|---|---|---|
| | | | (18:4/14:0 or 14:0/18:4) | | | | | 144.11 | |
| 23 | 11.47 | Chlorophyll | Pheophytin *c2* | $C_{35}H_{30}N_4O_5$ | 587.231 $[M+H]^+$ | 2.6 | 2 | 543.25, 527.22, 511.23 | 224, 450 |
| 24 | 11.47 | Phosphatidylcholine | | $C_{45}H_{82}NO_7P$ | 780.594 $[M+H]^+$ | 4.2 | 3 | 520.38, 502.37, 496.38, 478.36, 184.08 | |
| 25 | 11.55 | Betaine lipid | DGTSA 30:2 (16:2/14:0 or 14:0/16:2) | $C_{40}H_{73}NO_7$ | 680.551 $[M+H]^+$ | 6.6 | 2 | 470.35, 452.35, 446.36, 428.36, 144.10 | |
| 26 | 11.60 | Betaine lipid | DGTSA 34:5 (20:5/14:0 or 14:0/20:5) | $C_{44}H_{75}NO_7$ | 730.575 $[M+H]^+$ | 17.6 [f] | 2 | 520.38, 502.36, 446.36, 428.35, 144.10 | |
| 27 | 11.68 | Phosphatidylcholine | | | 806.611 $[M+H]^+$ | | 3 | 702.53, 522.39, 520.39, 504.38, 502.37, 184.08 | |
| 28 | 11.72 | Betaine lipid | DGTSA 32:3 (18:3/14:0 or 14:0/18:3) | $C_{42}H_{75}NO_7$ | 706.569 $[M+H]^+$ | 9.7 | 2 | 496.37, 478.36, 446.36, 428.35, 144.10 | |
| 29 | 11.80 | Phosphatidylcholine | | $C_{45}H_{84}NO_7P$ | 782.607 $[M+H]^+$ | 0.8 | 3 | 678.53, 184.08 | |
| 30 | 11.84 | Carotenoid | | $C_{40}H_{52}O_3$ | 581.401 $[M+H]^+$ | 2.6 | 3 | 563.42, 411.29, 355.26, 277.21, 251.20, 185.14, 127.12, 109.11 | 225, 265, 330, 442, 470 |
| 31 | 11.86 | Betaine lipid | DGTSA 28:0 (14:0/14:0) | $C_{38}H_{73}NO_7$ | 656.550 $[M+H]^+$ | 5.3 | 2 | 446.38, 428.37, 109.11 | |
| 32 | 11.91 | Betaine lipid | DGTSA 34:4 (20:4/14:0 or 14:0/20:4) | $C_{44}H_{77}NO_7$ | 732.589 $[M+H]^+$ | 15.2 [f] | 2 | 522.41, 504.40, 446.37, 428.37, 144.11 | |
| 33 | 12.08 | Betaine lipid | DGTSA 32:2 (18:2/14:0 or 14:0/18:2) | $C_{42}H_{77}NO_7$ | 708.593 $[M+H]^+$ | 21.4 [f] | 2 | 498.41, 480.40, 446.38, 428.37, 144.11 | |
| 34 | 12.09 | Phospholipid | | $C_{43}H_{84}NO_7P$ | 758.605 $[M+H]^+$ | 1.8 | 3 | 654.53, 520.37, 502.36, 474.40, 456.39, 184.08 | |
| 35 | 12.13 | Phospholipid | | $C_{45}H_{86}NO_7P$ | 784.623 $[M+H]^+$ | 1.3 | 3 | 647.54, 522.41, 504.39, 498.42, 480.40, 184.09, 144.11 | |
| 36 | 12.26 | Betaine lipid | | $C_{44}H_{79}NO_7$ | 734.605 $[M+H]^+$ | 15.7 | 3 | 524.43, 506.42, 496.40, 474.41, 456.40, 446.38, 428.36, 144.11 | |
| 37 | 12.30 | Phosphatidylethanolamine | | $C_{47}H_{88}NO_7P$ | 810.637 $[M+H]^+$ | 0.8 | 3 | 669.51, 522.41, 504.39, 164.02 | |
| 38 | 12.50 | Betaine lipid | DGTSA 30:0 (16:0/14:0 or 14:0/16:0) | $C_{40}H_{77}NO_7$ | 684.585 $[M+H]^+$ | 10.5 [f] | 2 | 474.40, 456.39, 446.37, 428.35 | |
| 39 | 12.56 | Betaine lipid | DGTSA 32:1 (18:1/14:0 or 14:0/18:1) | $C_{42}H_{79}NO_7$ | 710.599 $[M+H]^+$ | 7.8 | 2 | 500.42, 482.40, 446.36, 428.36, 144.11 | |
| 40 | 12.59 | Chlorophyll | Methylpheophorbide | $C_{36}H_{38}N_4O_5$ | 607.292 $[M+H]^+$ | 0.1 | 2 | 547.29 | |

| | | | | | | | | |
|---|---|---|---|---|---|---|---|---|
| | | | *a* | | | | | |
| 41 | 12.73 | Betaine lipid | | $C_{44}H_{81}NO_7$ | 736.619 $[M+H]^+$ | 13.4 [f] | 3 | 498.40, 480.38, 474.39, 456.38 |
| 42 | 13.26 | Tocopherol | $\alpha$-Tocopherol | $C_{29}H_{50}O_2$ | 429.364 $[M+H-H_2]^+$ | 21.6 [f] | 2 | 311.30, 205.12, 191.11, 165.09, 163.11 |
| 43 | 13.37 | Chlorophyll | Pheophytin *a* | $C_{55}H_{74}N_4O_5$ | 871.582 $[M+H]^+$ | 9.5 | 2 | 593.32, 533.29 |
| 44 | 13.59 | Carotenoid | Zeaxanthin | $C_{40}H_{56}O_2$ | 569.433 $[M+H]^+$ | 5.0 | 2 | 477.42 |

[a] Confidence level of the metabolite identification based on Sumner *et al*. 2007 and Blaženovic *et al*. 2018.

[b] When no $\lambda_{max}$ indicated, either no absorbance, or compound in too low amount to be detected

[c] LysoPC: lysophosphatidylcholine

[d] DGTSA: either DGTS [1,2-diacylglyceryl-*O*-2'-(hydroxymethyl)-(*N*,*N*,*N*-trimethyl)homoserine] or DGTA [1,2-diacylglyceryl-*O*-2'-(hydroxymethyl)-(*N*,*N*,*N*-trimethyl)-β-alanine]

[e] LysoPE: lysophosphatidylethanolamine

[f] High Δ ppm (≥ 10 ppm) due to a detection saturation or to a very low intensity of the ion in the UPLC-MS chromatogram

[g] Identification confirmed by comparison with a purchased commercial standard

**Table S2.** Dereplication table of the 54 compounds significantly varying with collection month.

| Box plot ID | Compound ID | RT (min) | Chemical class | Tentative identification | Formula (M) | MS[1] ($m/z$) | Error (Δ ppm) | Confidence level[a] | MS[2] ($m/z$) | UV $\lambda_{max}$ (nm)[b] |
|---|---|---|---|---|---|---|---|---|---|---|
| I | 1 | 0.98 | Phlorotannins | Fucodiphlorethol A, B, C, D, E, G or Difucophlorethol A or Tetrafucol A, B or Tetraphlorethol A, C, E | $C_{24}H_{18}O_{12}$ | 499.084 $[M+H]^+$ | 7.3 | 2 | 481.09, 463.08, 411.08, 395.09, 393.07, 355.06, 337.05, 327.06, 287.07, 285.05, 271.04, 269.05 139.04 | |
| II | | 5.23 | | | | 507.251 | | | | |
| III | | 6.19 | | | | 535.285 | | | | |
| IV | 3 | 6.27 | Betaine lipids | LysoDGTSA[c] 14:0 | $C_{24}H_{47}NO_6$ | 446.339 $[M+H]^+$ | 20.5[d] | 2 | 428.35, 285.25, 236.16, 144.11 | |
| V | 7 | 6.81 | Betaine lipids | LysoDGTSA 18:2 | $C_{28}H_{51}NO_6$ | 498.376 $[M+H]^+$ | 6.9 | 2 | 480.37, 361.29, 236.16, 144.11 | |
| VI | 10 | 7.45 | Betaine lipids | LysoDGTSA 16:0 | $C_{26}H_{51}NO_6$ | 474.373 $[M+H]^+$ | 13.6 [d] | 2 | 456.40, 351.14, 313.29, 236.16, 144.11 | |
| VII | 12 | 7.76 | Carotenoids | Fucoxanthin (dehydrated) | $C_{42}H_{58}O_6$ | 641.427 $[M+H-H_2O]^+$ | 10.0 [d] | 2 | 549.37, 221.16, 203.15 | |
| VIII | 14 | 9.30 | Chlorophylls | Chlorophyll *c2* | $C_{35}H_{28}MgN_4O_5$ | 609.204 $[M+H]^+$ | 8.5 | 2 | 591.23, 549.21, 534.23, 532.19 | |

| | | | | | | | | | |
|---|---|---|---|---|---|---|---|---|---|
| IX | | 9.37 | | | | 658.532 | | | 448.34, 446.36, 430.34, 428.35 | 224, 450 |
| X | | 9.61 | | | | 600.420 | | | 291.19, 263.20, 145.11, 121.11 | 224, 450 |
| XI | 15 | 9.61 | Chlorophylls | | | 623.413 $[M+H]^+$ | | 3 | 605.42, 587.42, 549.34, 531.34, 295.17, 259.13 | 224, 450 |
| XII | | 9.87 | | | | 768.576 | | | 750.57 | |
| XIII | | 10.25 | | | | 686.562 | | | | |
| XIV | 16 | 10.71 | Phosphatidylcholines | | | 782.590 $[M+H]^+$ | | 3 | 750.57, 572.39, 554.39, 446.37, 428.35, 184.08 | |
| XV | 18 | 10.93 | Carotenoids | Fucoxanthinol | $C_{40}H_{56}O_5$ | 599.408 $[M+H-H_2O]^+$ | 3.4 | 2 | 581.42, 411.28, 355.25, 277.21, 263.19, 213.13, 199.15, 185.14, 173.14, 127.11, 109.10 | 224, 265, 330, 447, 470 |

| | | | | | | | | | | |
|---|---|---|---|---|---|---|---|---|---|---|
| XVI | 19 | 10.94 | Carotenoids | Fucoxanthin | $C_{42}H_{58}O_6$ | 681.421 [M+Na]$^+$<br>659.435 [M+H]$^+$ | 11.6 [d]<br>6.4 | 1 [g]<br>1 | 663.42, 621.41, 603.40, 527.33<br>641.46, 581.43, 109.11 | 224, 265, 330, 447, 470 |
| XVII | 20 | 11.20 | Chlorophylls | Pheophorbide *a* | $C_{35}H_{36}N_4O_5$ | 593.279 [M+H]$^+$ | 4.4 | 2 | 533.27 | 224, 265, 330, 409, 505, 535 |
| XVIII | 21 | 11.35 | Phosphatidylcholines | | | 810.625 [M+H]$^+$ | | 3 | 778.60, 313.28, 184.08 | |
| XIX | 22 | 11.40 | Betaine lipids | DGTSA 32:4 (18:4/14:0 or 14:0/18:4) | $C_{42}H_{73}NO_7$ | 704.553 [M+H]$^+$ | 9.2 | 2 | 494.37, 476.35, 446.38, 428.35, 144.11 | |
| XX | 23 | 11.47 | Chlorophylls | Pheophytin *c2* | $C_{35}H_{30}N_4O_5$ | 587.231 [M+H]$^+$ | 2.6 | 2 | 543.25, 527.22, 511.23 | 224, 450 |
| XXI | 24 | 11.47 | Phosphatidylcholines | | $C_{45}H_{82}NO_7P$ | 780.594 [M+H]$^+$ | 4.2 | 3 | 520.38, 502.37, 496.38, 478.36, 184.08 | |
| XXII | 25 | 11.55 | Betaine lipids | DGTSA 30:2 (16:2/14:0 or 14:0/16:2) | $C_{40}H_{73}NO_7$ | 680.551 [M+H]$^+$ | 6.6 | 2 | 470.35, 452.35, 446.36, 428.36, 144.10 | |
| XXIII | 26 | 11.60 | Betaine lipids | DGTSA 34:5 (20:5/14:0 or 14:0/20:5) | $C_{44}H_{75}NO_7$ | 730.575 [M+H]$^+$ | 17.6 [d] | 2 | 520.38, 502.36, 446.36, 428.35, 144.10 | |
| XXIV | 27 | 11.68 | Phosphatidylcholines | | | 806.611 | | 3 | 702.53, | |

| | | | | | | | | | | |
|---|---|---|---|---|---|---|---|---|---|---|
| | | | | | | $[M+H]^+$ | | | 522.39, 520.39, 504.38, 502.37, 184.08 | |
| XXV | 28 | 11.72 | Betaine lipids | DGTSA 32:3 (18:3/14:0 or 14:0/18:3) | $C_{42}H_{75}NO_7$ | 706.569 $[M+H]^+$ | 9.7 | 2 | 496.37, 478.36, 446.36, 428.35, 144.10 | |
| XXVI | 29 | 11.80 | Phosphatidylcholines | | $C_{45}H_{84}NO_7P$ | 782.607 $[M+H]^+$ | 0.8 | 3 | 678.53, 184.08 | |
| XXVII | 30 | 11.84 | Carotenoids | | $C_{40}H_{52}O_3$ | 581.401 $[M+H]^+$ | 2.6 | 3 | 563.42, 411.29, 355.26, 277.21, 251.20, 185.14, 127.12, 109.11 | 225, 265, 330, 442, 470 |
| XXVIII | | 11.86 | | | | 641.427 | | | 581.42, 411.29, 251.20, 109.10 | 225, 330, 442 |
| XXIX | | 11.86 | | | | 681.415 | | | 663.42, 603.39, 527.33 | 225, 330, 442 |
| XXX | 31 | 11.86 | Betaine lipids | DGTSA 28:0 (14:0/14:0) | $C_{38}H_{73}NO_7$ | 656.550 $[M+H]^+$ | 5.3 | 2 | 446.38, 428.37, 109.11 | |
| XXXI | 32 | 11.91 | Betaine lipids | DGTSA 34:4 (20:4/14:0 or 14:0/20:4) | $C_{44}H_{77}NO_7$ | 732.589 $[M+H]^+$ | 15.2 [d] | 2 | 522.41, 504.40, 446.37, 428.37, 144.11 | |
| XXXII | | 11.93 | | | | 682.569 | | | 603.41, 527.34 | 225, 265, 330, 442, 470 |

| | | | | | | | | | |
|---|---|---|---|---|---|---|---|---|---|
| XXXIII | | 11.95 | | | | 808.623 | | | 704.58, 522.40, 504.38, 377.33 |
| XXXIV | | 12.01 | | | | 710.593 | | | 498.40, 480.39, 446.37, 428.35, 144.11 |
| XXXV | | 12.05 | | | | 708.793 | | | |
| XXXVI | 33 | 12.08 | Betaine lipids | DGTSA 32:2 (18:2/14:0 or 14:0/18:2) | $C_{42}H_{77}NO_7$ | 708.593 $[M+H]^+$ | 21.4 [d] | 2 | 498.41, 480.40, 446.38, 428.37, 144.11 |
| XXXVII | 35 | 12.13 | Phospholipids | | $C_{45}H_{86}NO_7P$ | 784.623 $[M+H]^+$ | 1.3 | 3 | 647.54, 522.41, 504.39, 498.42, 480.40, 184.09, 144.11 |
| XXXVIII | | 12.15 | | | | 333.223 | | | 241.21, 161.14, 145.11, 133.11, 121.11, 119.09, 107.09, 105.07, 95.09, 93.07, 91.06, 81.07 |
| XXXIX | | 12.16 | | | | 635.473 | | | 617.48, 543.44, 359.27, 285.23, 267.22, 241.20, 201.17, 161.14, |

| | | | | | | | | | | |
|---|---|---|---|---|---|---|---|---|---|---|
| | | | | | | | | | 147.12, 133.16 | |
| XL | | 12.17 | | | | 976.626 | | | | 225 |
| XLI | 36 | 12.26 | Betaine lipids | | $C_{44}H_{79}NO_7$ | 734.605 [M+H]+ | 15.7 | 3 | 524.43, 506.42, 496.40, 474.41, 456.40, 446.38, 428.36, 144.11 | |
| XLII | 37 | 12.30 | Phosphatidylethanolamines | | $C_{47}H_{88}NO_7P$ | 810.637 [M+H]+ | 0.8 | 3 | 669.51, 522.41, 504.39, 164.02 | |
| XLIII | | 12.31 | | | | 957.580 | | | 795.53, 679.36 | |
| XLIV | | 12.48 | | | | 760.623 | | | 697.31, 656.55, 522.4, 504.39, 474.40, 456.38, 236.57, 144.11 | |
| XLV | 38 | 12.50 | Betaine lipids | DGTSA 30:0 (16:0/14:0 or 14:0/16:0) | $C_{40}H_{77}NO_7$ | 684.585 [M+H]+ | 10.5 [d] | 2 | 474.40, 456.39, 446.37, 428.35 | |
| XLVI | 39 | 12.56 | Betaine lipids | DGTSA 32:1 (18:1/14:0 or 14:0/18:1) | $C_{42}H_{79}NO_7$ | 710.599 [M+H]+ | 7.8 | 2 | 500.42, 482.40, 446.36, 428.36, 144.11 | |
| XLVII | 40 | 12.59 | Chlorophylls | Methylpheophorbide *a* | $C_{36}H_{38}N_4O_5$ | 607.292 [M+H]+ | 0.1 | 2 | 547.29 | |
| XLVIII | | 12.66 | | | | 983.600 | | | 705.37, 681.37 | 225 |
| XLIX | | 12.69 | | | | 767.482 | | | 519.32, 489.27 | |
| L | 41 | 12.73 | Betaine lipids | | $C_{44}H_{81}NO_7$ | 736.619 | 13.4 [d] | 3 | 498.40, | |

| | | | | |
|---|---|---|---|---|
| | | $[M+H]^+$ | 480.38, 474.39, 456.38 | |
| LI | 12.84 | 793.501 | 517.30 | |
| LII | 12.94 | 762.639 | 658.56, 520.48, 506.40, 474.41, 456.38, 144.09 | |
| LIII | 13.13 | 635.473 | | 200, 225, 450, 475 |
| LIV | 13.29 | 795.519 | 517.30 | |

[a] Confidence level of the metabolite identification based on Sumner *et al*. 2007 and Blaženovic *et al*. 2018.

[b] When no $\lambda_{max}$ indicated, either no absorbance, or compound in too low amount to be detected

[c] DGTSA: either DGTS [1,2-diacylglyceryl-*O*-2'-(hydroxymethyl)-(*N*,*N*,*N*-trimethyl)homoserine] or DGTA [1,2-diacylglyceryl-*O*-2'-(hydroxymethyl)-(*N*,*N*,*N*-trimethyl)-β-alanine]

[d] High Δ ppm (≥ 10 ppm) due to a detection saturation or to a very low intensity of the ion in the UPLC-MS chromatogram

**Table S3.** Pathogen strains and their strain-specific culture conditions.

| Pathogen | Strain | Medium | Positive control | Pre-culture incubation temperature (°C) | Start optical density | Incubation temperature (°C) | Incubation time (h) |
|---|---|---|---|---|---|---|---|
| *Enterococcus faecium* | DSM 20478) | M92[a] | Ampicillin | 37 | 0.01 | 37 | 4 |
| Methicillin-resistant *Staphylococcus aureus* (MRSA) | DSM 18827 | TSB12[b] | Chloramphenicol | 28 | 0.01 | 37 | 5 |
| *Klebsiella pneumoniae* | DSM 30104 | TSB12[b] | Chloramphenicol | 28 | 0.01 | 37 | 5 |
| *Acinetobacter baumannii* | DSM 30007 | TSB12[b] | Ampicillin | 28 | 0.01 | 37 | 5 |
| *Pseudomonas aeruginosa* | DSM 1128 | TSB12[b] | Chloramphenicol | 37 | 0.01 | 37 | 5 |
| *Escherichia coli* | DSM 1576 | TSB12[b] | Chloramphenicol | 28 | 0.01 | 37 | 5 |
| *Candida albicans* | DSM 1386 | M186[c] | Nystatin | 28 | 0.03 | 37 | 5 |
| *Cryptococcus neoformans* | DSM 6973 | M186[c] | Amphotericin B | 28 | 0.03 | 28 | 6-7 |
| *Vibrio anguillarum* | DSM 21597 | TM[d] | Chloramphenicol | 28 | 0.01 | 28 | 5 |
| *Pseudoalteromonas bacteriolytica* | CIP 105725 | TM[d] | Chloramphenicol | 28 | 0.03 | 28 | 6-7 |
| *Pseudoalteromonas elyakovii* | CIP 105338 | TM[d] | Chloramphenicol | 28 | 0.03 | 28 | 6-7 |

[a] 30 g tryptic soy broth, 3 g yeast extract, 1000 mL $H_2O$, pH 7.0-7.2

[b] 12 g tryptic soy broth, 5 g NaCl, 1000 mL $H_2O$

[c] 10 g glucose, 5 g peptone from soybeans, 3 g yeast extract, 3 g malt extract, 1000 mL $H_2O$

[d] 5 g peptone from soybeans, 1 g yeast extract, 30 g Tropic Marin artificial sea salt, 1000 mL $H_2O$

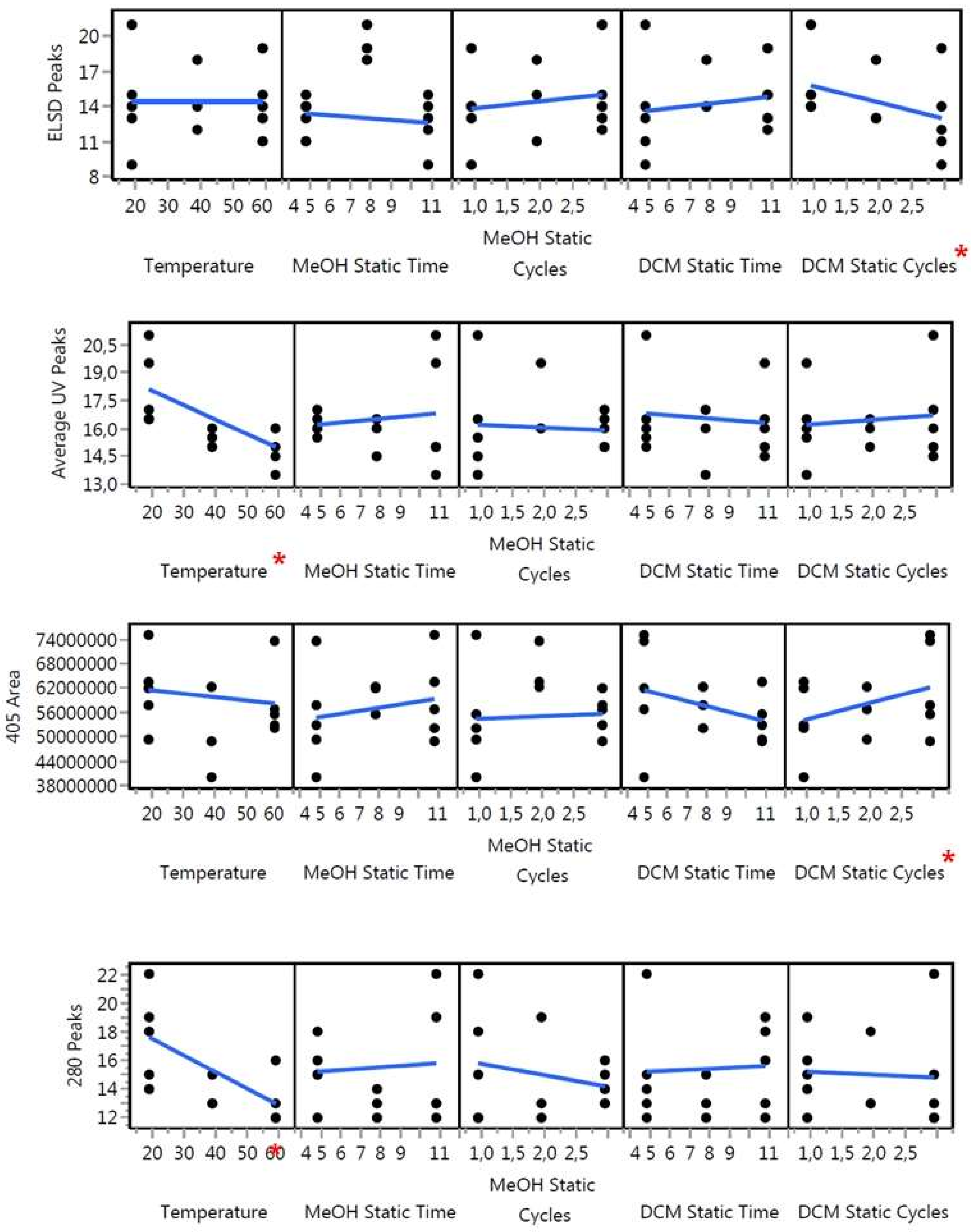


**Figure S1.** Statistical linear regression analyses of the relationships between the five extraction variables and: (**A**) number of ELSD signal peaks (1st repeat); (**B**) average number of UV peaks (2nd repeat); (**C**) 405 nm UV peak area (2nd repeat); (**D**) number of 280 nm UV peaks (2nd repeat).

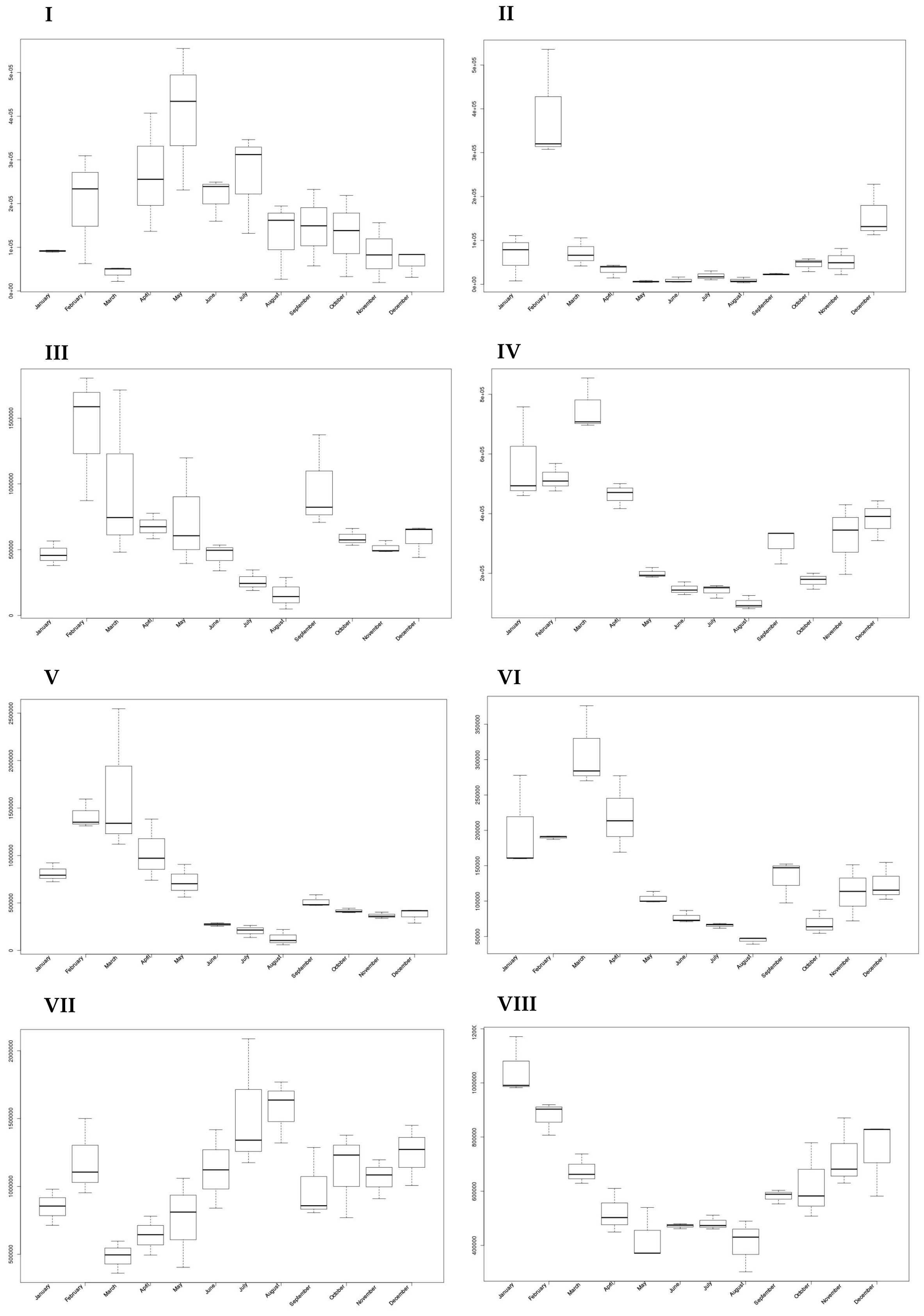


**Figure S2.** Seasonality box plots depicting monthly median (bold), 1st-3rd quartiles (lower-upper box limits), and minimum and maximum (lower and upper bars) total ion count for compounds I-VIII.

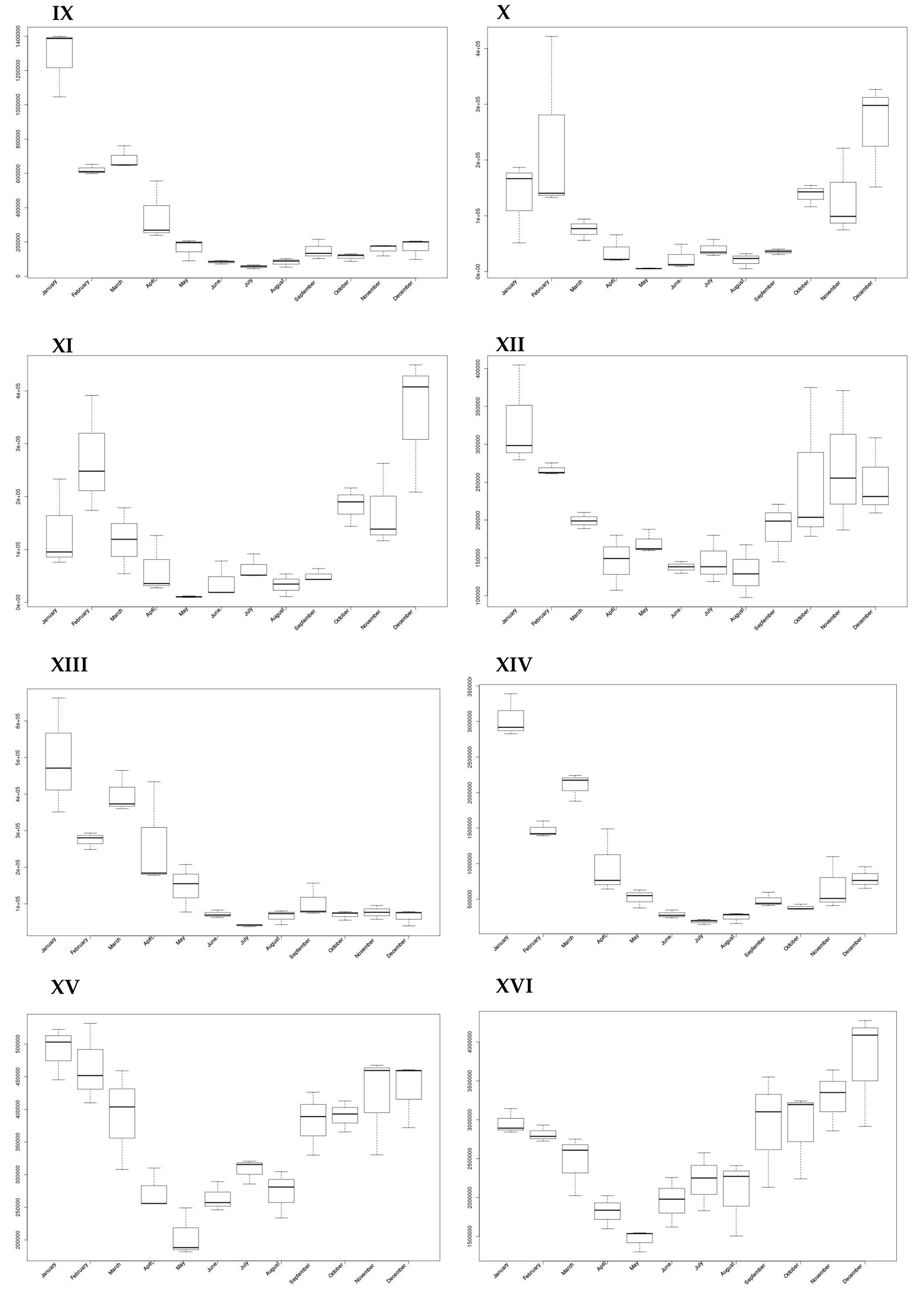


**Figure S3.** Seasonality box plots depicting monthly median (bold), 1st-3rd quartiles (lower-upper box limits), and minimum and maximum (lower and upper bars) total ion count for compounds IX-XVI.

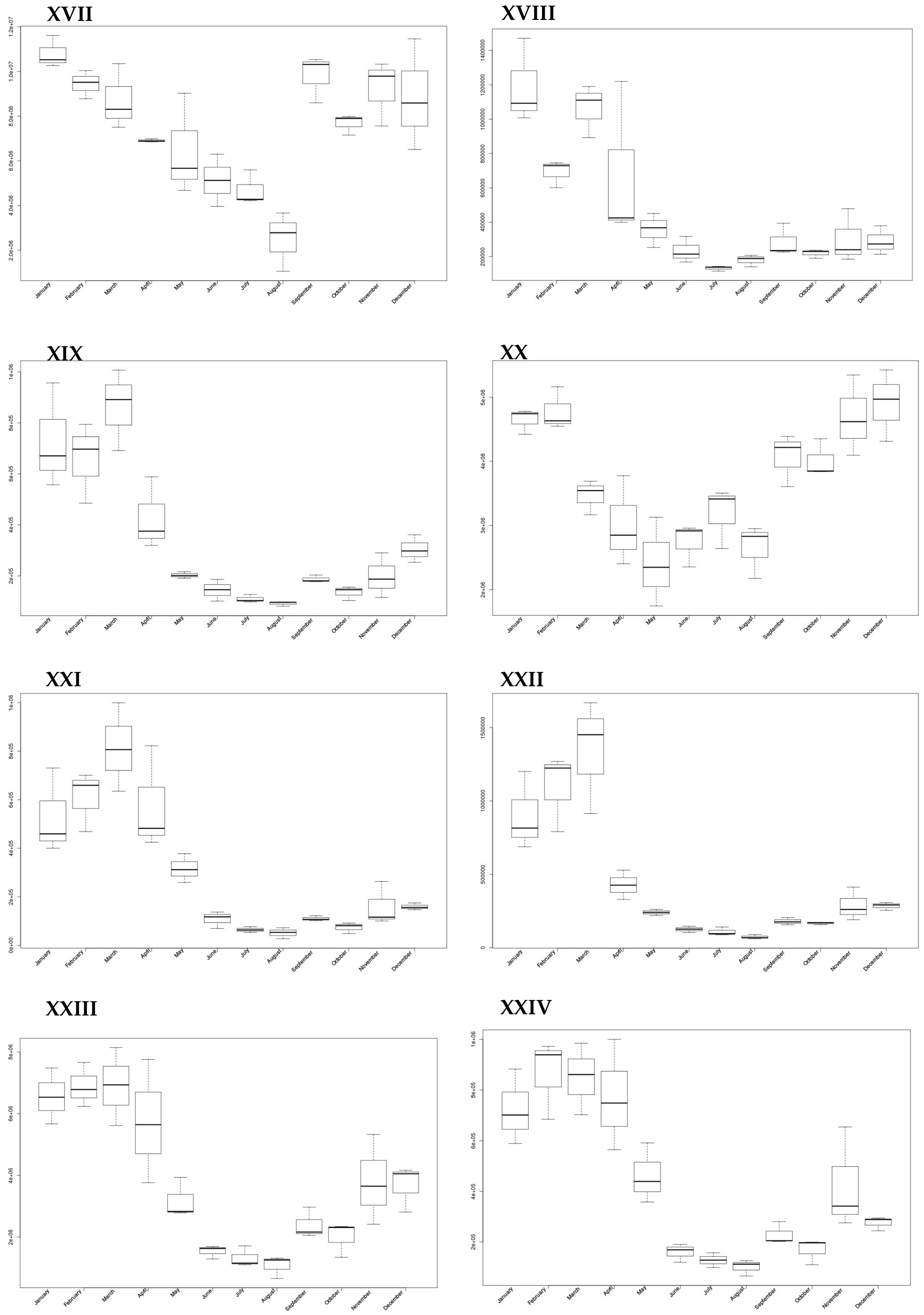


**Figure S4.** Seasonality box plots depicting monthly median (bold), 1st-3rd quartiles (lower-upper box limits), and minimum and maximum (lower and upper bars) total ion count for compounds XVII-XXIV.

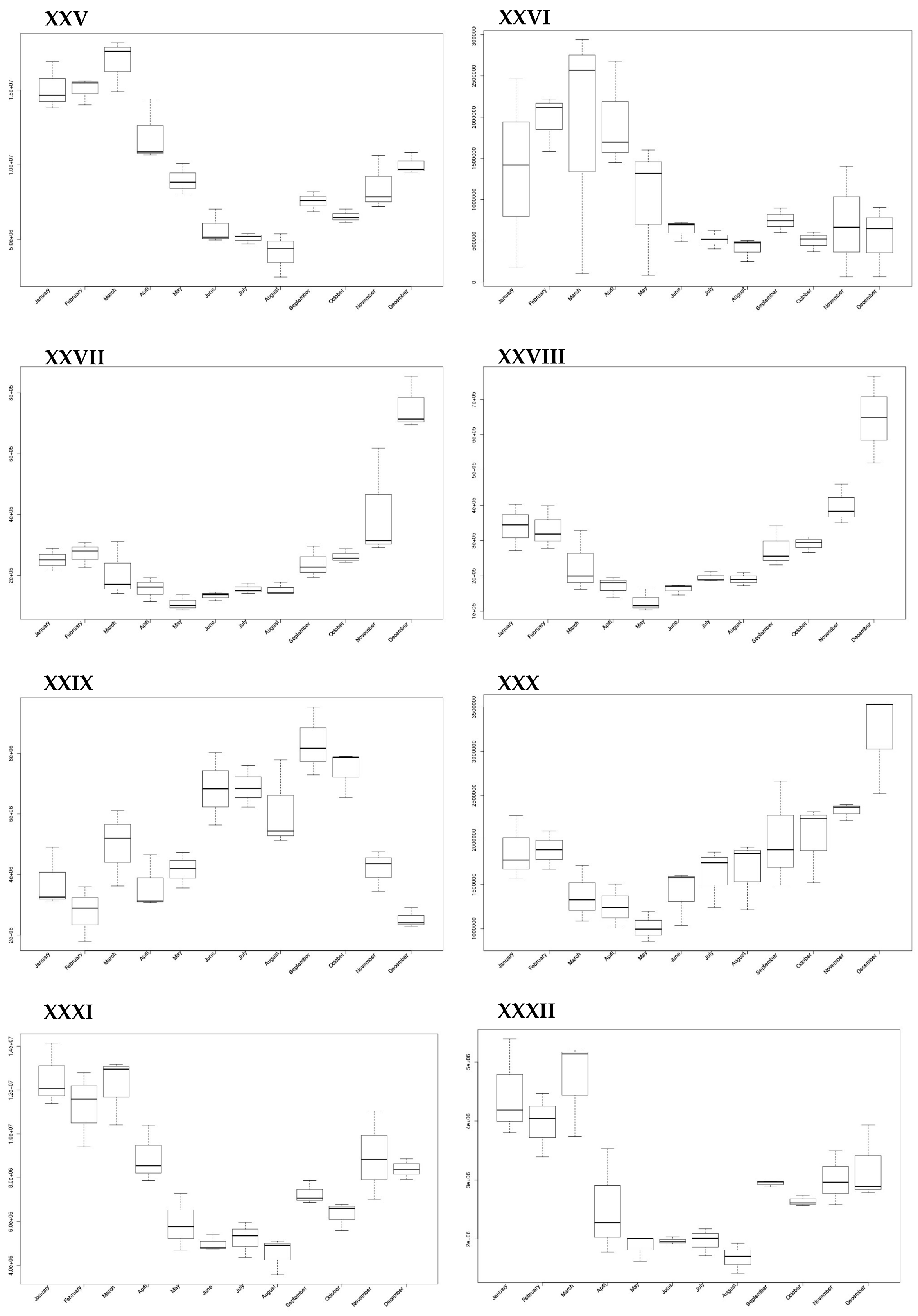


**Figure S5.** Seasonality box plots depicting monthly median (bold), 1st-3rd quartiles (lower-upper box limits), and minimum and maximum (lower and upper bars) total ion count for compounds XXV-XXXII.

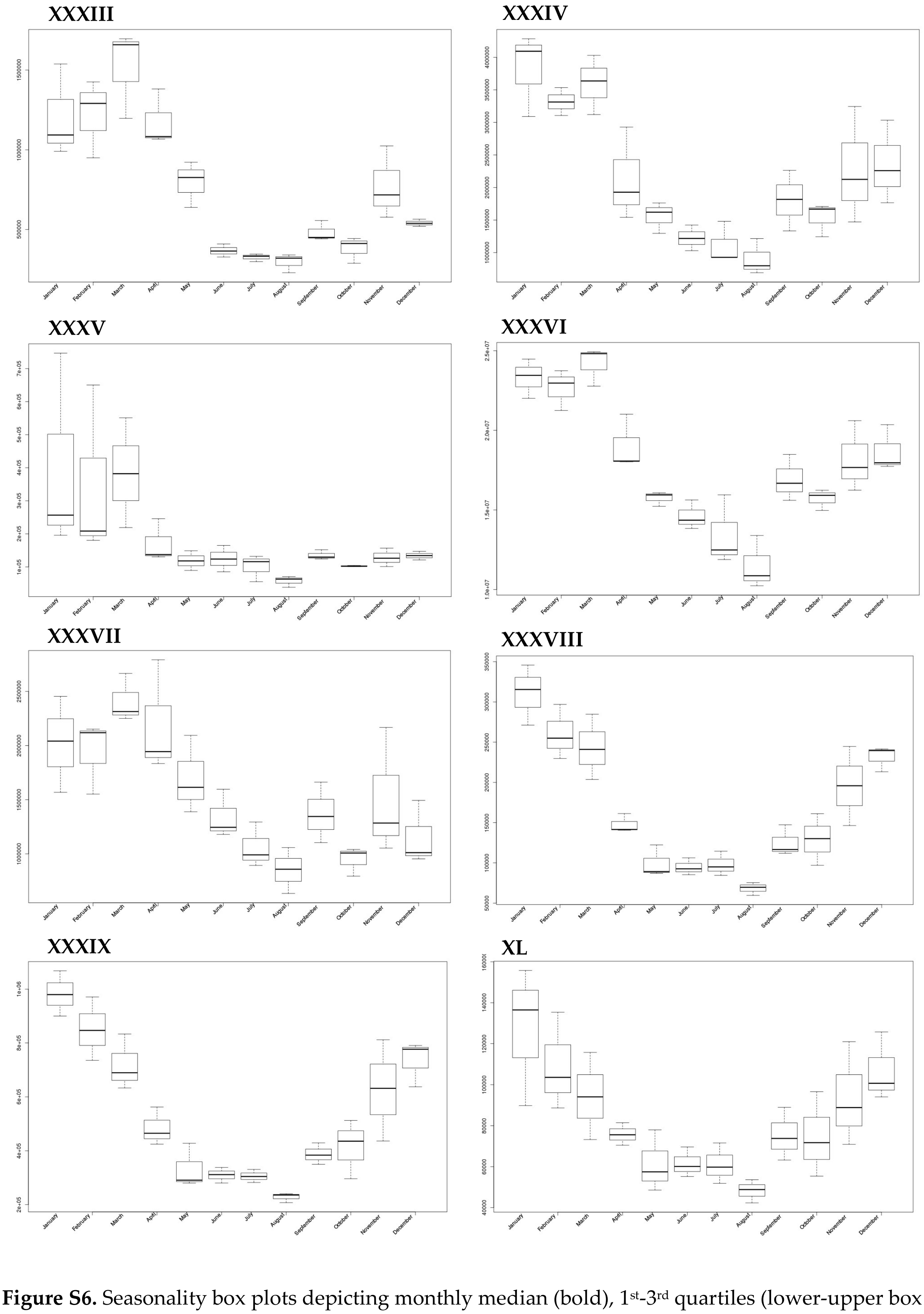


**Figure S6.** Seasonality box plots depicting monthly median (bold), 1st-3rd quartiles (lower-upper box limits), and minimum and maximum (lower and upper bars) total ion count for compounds XXXIII-XL.

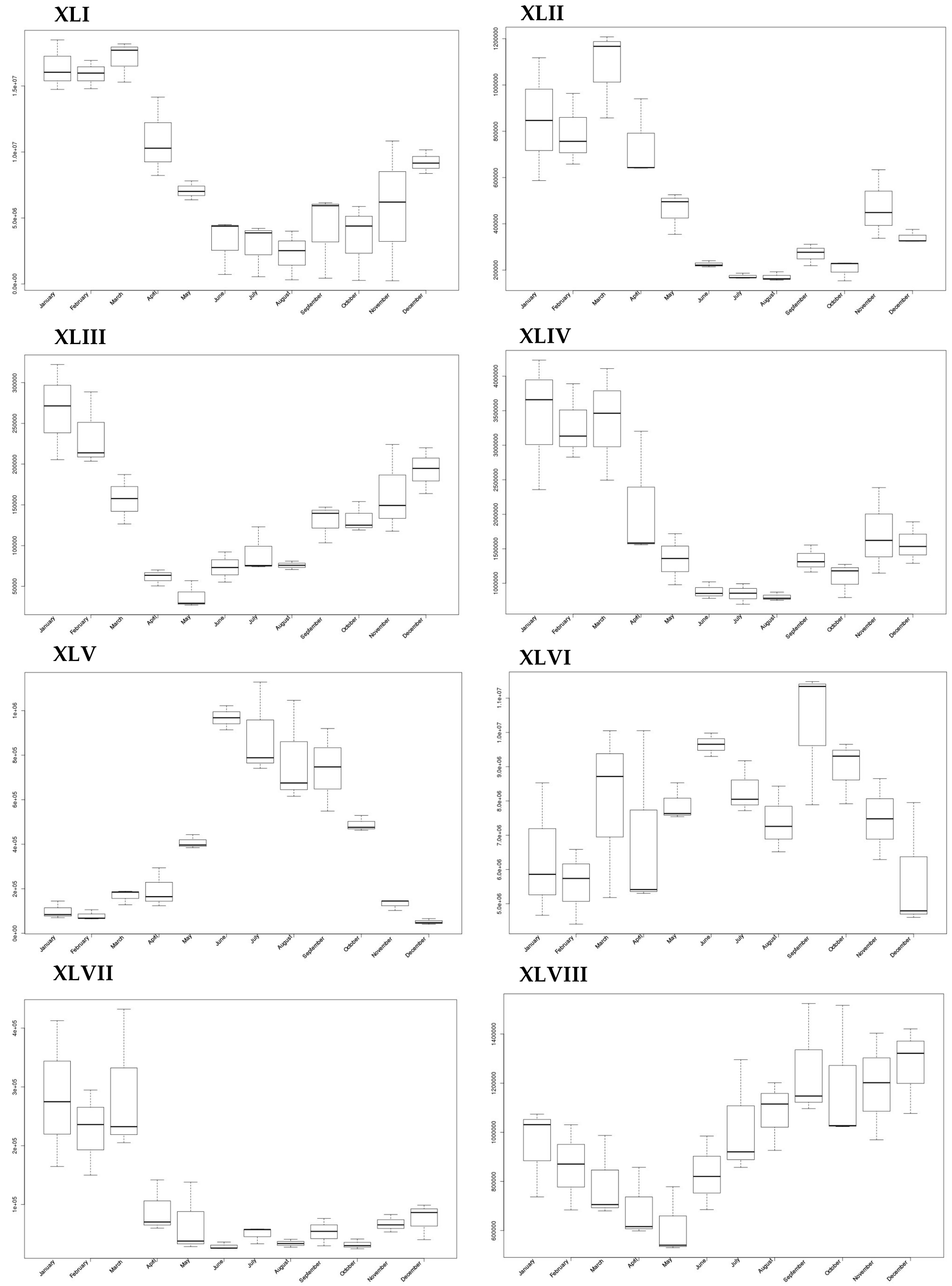


**Figure S7.** Seasonality box plots depicting monthly median (bold), 1st-3rd quartiles (lower-upper box limits), and minimum and maximum (lower and upper bars) total ion count for compounds XLI-XLVIII.

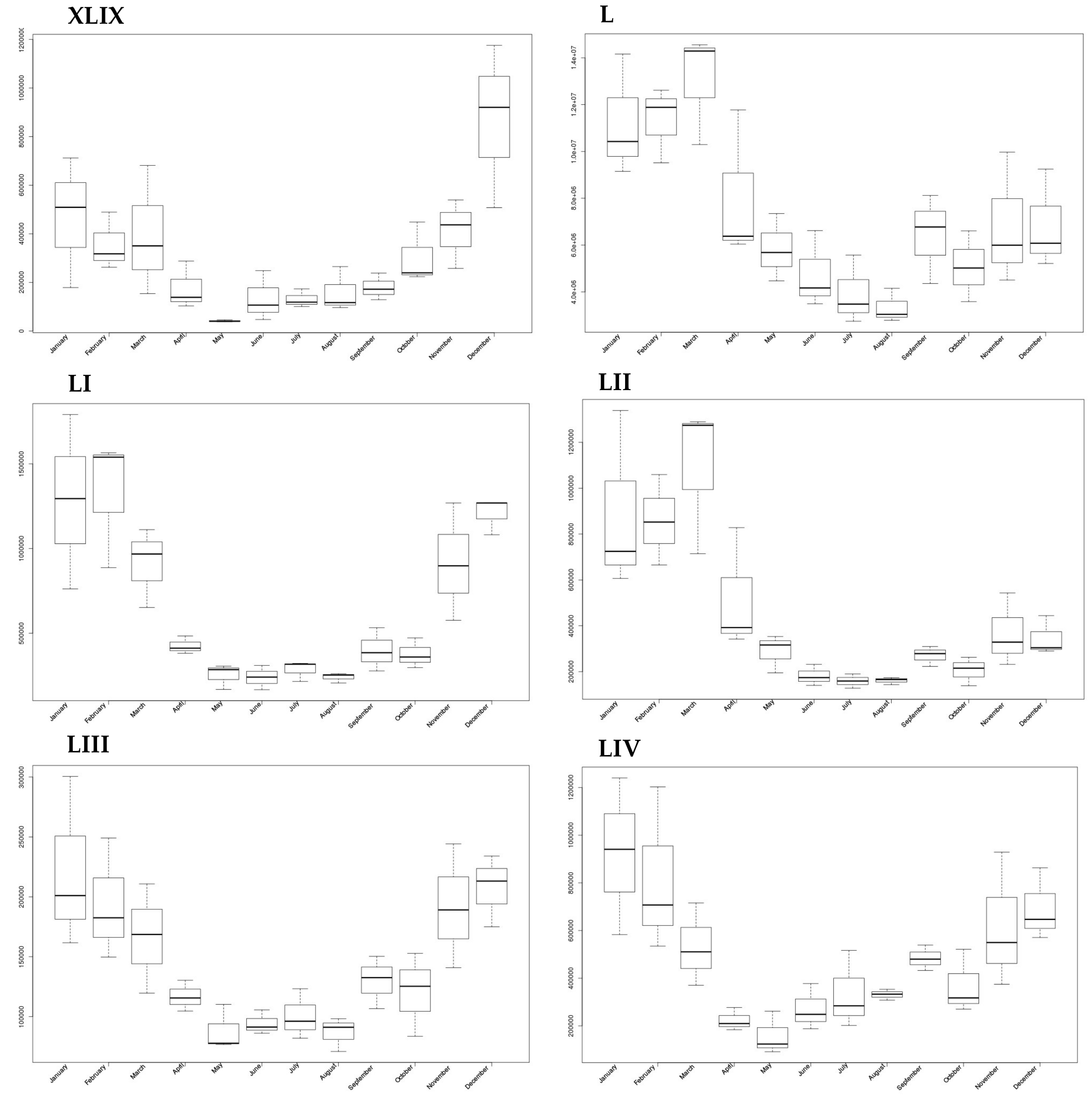


**Figure S8.** Seasonality box plots depicting monthly median (bold), 1st-3rd quartiles (lower-upper box limits), and minimum and maximum (lower and upper bars) total ion count for compounds XLIX-LIV.
